# Detecting Critical Functional Ingredients Group and Mechanism of Xuebijing Injection in Treating Sepsis

**DOI:** 10.3389/fphar.2021.769190

**Published:** 2021-12-06

**Authors:** Qi- Wu, Chuan-hui Yin, Yi Li, Jie-qi Cai, Han-yun Yang, Ying-ying Huang, Yi-xu Zheng, Ke Xiong, Hai-lang Yu, Ai-ping Lu, Ke-xin Wang, Dao-gang Guan, Yu-peng Chen

**Affiliations:** ^1^ Department of Burns, Nanfang Hospital, Southern Medical University, Guangzhou, China; ^2^ Department of Biochemistry and Molecular Biology, School of Basic Medical Sciences, Southern Medical University, Guangzhou, China; ^3^ Guangdong Province Key Laboratory of Single Cell Technology and Application, Southern Medical University, Guangzhou, China; ^4^ Department of Radiology, Nanfang Hospital, Southern Medical University, Guangzhou, China; ^5^ The First Clinical Medical College of Southern Medical University, Guangzhou, China; ^6^ Department of Obstetrics and Gynecology, Nanfang Hospital, Southern Medical University, Guangzhou, China; ^7^ Department of Ophthalmology, Nanfang Hospital, Southern Medical University, Guangzhou, China; ^8^ Institute of Integrated Bioinformedicine and Translational Science, Hong Kong Baptist University, Kowloon Tong, Hong Kong China; ^9^ National Key Clinical Specialty/Engineering Technology Research Center of Education Ministry of China, Guangdong Provincial Key Laboratory on Brain Function Repair and Regeneration, Department of Neurosurgery, Neurosurgery Institute, Zhujiang Hospital, Southern Medical University, Guangzhou, China

**Keywords:** Xuebijing injection (XBJI), sepsis, network pharmacology, functional response space, critical functional ingredients group, key response proteins

## Abstract

Sepsis is a systemic inflammatory reaction caused by various infectious or noninfectious factors, which can lead to shock, multiple organ dysfunction syndrome, and death. It is one of the common complications and a main cause of death in critically ill patients. At present, the treatments of sepsis are mainly focused on the controlling of inflammatory response and reduction of various organ function damage, including anti-infection, hormones, mechanical ventilation, nutritional support, and traditional Chinese medicine (TCM). Among them, Xuebijing injection (XBJI) is an important derivative of TCM, which is widely used in clinical research. However, the molecular mechanism of XBJI on sepsis is still not clear. The mechanism of treatment of “bacteria, poison and inflammation” and the effects of multi-ingredient, multi-target, and multi-pathway have still not been clarified. For solving this issue, we designed a new systems pharmacology strategy which combines target genes of XBJI and the pathogenetic genes of sepsis to construct functional response space (FRS). The key response proteins in the FRS were determined by using a novel node importance calculation method and were condensed by a dynamic programming strategy to conduct the critical functional ingredients group (CFIG). The results showed that enriched pathways of key response proteins selected from FRS could cover 95.83% of the enriched pathways of reference targets, which were defined as the intersections of ingredient targets and pathogenetic genes. The targets of the optimized CFIG with 60 ingredients could be enriched into 182 pathways which covered 81.58% of 152 pathways of 1,606 pathogenetic genes. The prediction of CFIG targets showed that the CFIG of XBJI could affect sepsis synergistically through genes such as TAK1, TNF-α, IL-1β, and MEK1 in the pathways of MAPK, NF-κB, PI3K-AKT, Toll-like receptor, and tumor necrosis factor signaling. Finally, the effects of apigenin, baicalein, and luteolin were evaluated by *in vitro* experiments and were proved to be effective in reducing the production of intracellular reactive oxygen species in lipopolysaccharide-stimulated RAW264.7 cells, significantly. These results indicate that the novel integrative model can promote reliability and accuracy on depicting the CFIGs in XBJI and figure out a methodological coordinate for simplicity, mechanism analysis, and secondary development of formulas in TCM.

## 1 Introduction

Sepsis is a systemic inflammatory response syndrome (SIRS) caused by bacteria and other pathogenic microorganisms invading the body, which can lead to shock, multiple organ dysfunction syndrome (MODS), and death. It is one of the common complications of critical patients and the main cause of death in critically ill patients (Cuthbertson et al., 2013; Czupryna et al., 2013; Rhodes et al., 2017). A recent study estimated that the annual incidence of sepsis in the world increased to about 48.9 million, of which 11 million died, accounting for 19.70% of the total deaths in the world (Rudd et al., 2020). It is a complex and thorny problem in modern intensive care medicine, which has already posed a serious threat to human health and brought a huge burden to economic development (Liu et al., 2014; Liang et al., 2020). Therefore, it is of great theoretical value and social significance to strengthen the basic research and clinical research on prevention and treatment of sepsis.

The pathogenesis of sepsis is complex and closely related to the pathophysiological changes of multiple systems and organs. It involves systemic inflammatory network effect, immune dysfunction, and abnormal response of host to different infectious pathogenic microorganisms and microbial toxins. According to the current mainstream views, the essence of sepsis is SIRS. Furthermore, sepsis is also treated as a cytokine storm which generates during various systemic acute infections (Kumar, 2020b; Karbian et al., 2020). It can include multi-organ failure or MODS and death of the patient. Nowadays, many articles focus on Toll-like receptor (TLR) signaling pathways in the cytokine storm which are generated during sepsis and in the host-derived endogenous molecules and factors which are involved in the negative regulation of TLR signaling (Kumar, 2020b). For example, TLR4 was reported to control bacterial clearance and the induction of pro-inflammatory immune response during bacterial sepsis (Deng et al., 2013). TLR2 and TLR4 had a more dynamic expression on the neutrophils of sepsis than on monocytes (Salomão et al., 2008). The expressions of TLR2, TLR3, TLR4, and TLR7 increased in the kidneys and intestine of sepsis mice, indicating that these four cytokines could influence the effect of pro-inflammatory response during infection (Krivan et al., 2019). High mobility group box 1 (HMGB1), a critical cytokine mediating organ damage, was a target of Xuebijing injection (XBJI) and could be regulated by 18 ml/kg Xuebijing (XBJ) to prevent MODS (Hutchins et al., 2014; Chen et al., 2016). During sepsis, strong inflammatory response could cause microvascular endothelial damage, sustained hypotension, and organ failure and might further lead to life-threatening organ dysfunction (Ehrman et al., 2018). During sepsis, pro-inflammatory/anti-inflammatory imbalance was an important factor leading to inflammatory reactions (Tsirigotis et al., 2015). IL-1β, IL-8, and TNF-α were typical pro-inflammatory factors, which could promote T cells to further produce a large number of inflammatory mediators, could reduce the body's immune function and destroy the barrier function of various tissues and organs, and could lead to the spread of systemic inflammatory response (Kamisoglu et al., 2015). Comparatively, IL-1Ra, IL-4, IL-6, IL-10, IL-11, IL-13, IL-35, and TGF-β are some anti-inflammatory cytokines (Chaudhry et al., 2013). Furthermore, some signaling pathways have been reported to relate with sepsis closely, such as the JAK/STAT signaling pathway could mediate the myocardial injury of septic rats (Jin et al., 2020); the PI3K-Akt signaling pathway could attenuate apoptosis and improve the survival in animal models of sepsis (Liu et al., 2019; Mizuta et al., 2020); the T-cell receptor signaling pathway could release some activated T cells and nuclear factors, CD4^+^, CD8^+^, and nuclear factor of activated T cells 2 (NFATC2), to take the immune response against sepsis (Beckmann et al., 2020; Kim et al., 2021); the JAK2/STAT3 signaling pathway could promote blood–brain barrier (BBB) impairment in rats with sepsis (Chen et al., 2020); the TLR signaling pathway could play a crucial role in sepsis-induced acute cardiac injury and acute injury of the lungs, kidneys, and intestine (Krivan et al., 2019; Kumar, 2020a; Lackner et al., 2020); the NF-κB/TNF-α signaling pathway could regulate inflammatory apoptosis (Chand et al., 2018) and alleviate the injury of lungs and intestinal barrier in sepsis (Wang et al., 2020; Cao et al., 2021). NF-κB could activate the cytokine storm by promoting the transcription of some pro-inflammatory cytokines, including IL-1β, IL-12, and TNF-α (Hotchkiss et al., 2016).

The application of traditional Chinese medicine (TCM) in disease treatment has been gradually accepted, especially some TCM injections. Some of its mechanisms in treating disease have gradually been analyzed. For example, Shenfu injection has been proved to attenuate apoptosis and inflammation induced by lipopolysaccharide (LPS) *via* downregulating the ERK and MEK signaling pathways (Chen et al., 2020); refined Qingkailing injection has been proved to inhibit inflammatory response against ischemic stroke by activating the AKT/PI3K signaling pathway, decreasing chemokines Ccl2, Cxcl2, Cxcl3, and pro-inflammatory factors tumor necrosis factor (TNF), IL-6, and IL-1b, and by increasing anti-inflammatory cytokine IL-10 (Ma et al., 2020). Re-Du-Ning injection (RDN) has been proved to ameliorate LPS-induced acute lung injury by downregulating some pro-inflammatory cytokines, such as IL-1β, IL-6, and TNF-α, and by suppressing the MAPK pathway (Yang et al., 2021).

Currently, the treatment of sepsis focuses on controlling inflammatory reactions and reducing the damage to various organs, mainly including anti-infection, hormones, mechanical ventilation, nutritional support, and TCM. Among these, statins are anti-infective drugs and belong to hydroxymethylglutarate coenzyme A reductase inhibitors and are currently widely used in improving the prognosis of septic patients (Falagas et al., 2008). These could improve the function of vascular endothelial cells, regulate cholesterol metabolism, decrease blood lipids, and reduce the release of effector cytokines by inhibiting cell signal transduction, thereby reducing the body's inflammatory response. Furthermore, some antibiotics have also been used in the treatment of sepsis with their immunomodulatory properties, for example, vancomycin and daptomycin (Muenster et al., 2021).

Comparatively, TCM has a good effect in treating sepsis, with mortality of sepsis to a certain extent. TCM treatment methods include “three syndromes and three methods,” namely, a method of activating blood to remove blood stasis in treating blood stasis syndrome, heat clearing, and detoxification in treating toxic heat syndrome, strengthening the body in treating acute deficiency syndrome, and including the treatment strategy of integrated TCM and Western medicine. Among the Chinese medicines, XBJI plays an important role in treating sepsis at present (Shao et al., 2011a; Shi et al., 2017). Nowadays, XBJI has been proved to stimulate the differentiation of Treg and inhibit the differentiation of Th17, moderately, during the cytokine storm in septic shock (Chen et al., 2018). These results proved that differentiation of Th17 could be inhibited by XBJI through the normalization of IL-17 expression, thereby improving the survival in sepsis mice (Joshi et al., 2012). The NF-κB signaling pathway could activate some pro-inflammatory cytokines, including IL-6 and TNF-α, and has been reported to be regulated by XBJI (Chen et al., 2018). Furthermore, the expression of some proteins in NF-κB signaling, such as CD14, CXCL2, and Ptgs2, has also been reported to be inhibited by XBJI (Wang et al., 2020). Furthermore, the five botanical drugs that comprise XBJI were reported to have a close relationship with sepsis or anti-inflammatory response. These five botanical drugs are *Carthamus tinctorius* L. (Honghua, HH), *Salvia miltiorrhiza* Bunge (Danshen, DS), *Paeonia lactiflora* Pall. (Chishao, CS), *Ligusticum striatum* DC (Chuanxiong, CX), and *Angelica sinensis* (Oliv.) Diels (Danggui, DG). According to the TCM theory, XBJI has an effect in clearing away heat, detoxifying, promoting blood circulation, and removing blood stasis. Among the composition of the botanical drugs in XBJI, HH played a primary role by activating blood circulation (Gong et al., 2015; Li et al., 2021) and removing blood stasis, and has been proved to mediate a pro-angiogenic role by enhancing the stability of VEGF-A and MMP-9 mRNA (Zou et al., 2018) and protecting LPS-induced cardiac fibrosis through the ERK1/2 signaling pathway while severe and potentially fatal hypotension and cardiac contractile dysfunction are treated as the common symptoms in patients with sepsis (Han et al., 2017). CS and CX play accessory roles in cooling the blood, removing blood stasis, and playing roles in alleviating, detoxifying, and magnifying the effects of the primary drug (Chen et al., 2011; Li et al., 2021). CS has been reported to inhibit inflammation in experimental sepsis and RAW264.7 cells (Jiang et al., 2009; Wang et al., 2014), improve survival in LPS-challenged mice by its important ingredient, paeoniflorin (Cao et al., 2011), and prevent acute lung injury induced by LPS through the PTEN/AKT pathway in a silencing information regulator 2–related enzyme 1–dependent manner by its important ingredient, oxypaeoniflorin (Guohua et al., 2021). CX has been indicated to inhibit LPS-induced IL-8 production in human umbilical vein endothelial cells at both the mRNA and protein levels (Li et al., 2009), suppress the production of nitric oxide (NO) and prostaglandin E2 in LPS-stimulated RAW264.7 cells through the NF-κB signaling pathways (Liu et al., 2017), increase superoxide dismutase (SOD) activity and eNOS mRNA, and decrease MDA content, myeloperoxidase (MPO) activity, and IL-1β (Lv et al., 2012). DS and DG are facilitatory drugs that enrich the blood and spread stasis (Li et al., 2021). DS has been reported to play a role in exhibiting significant anti-inflammatory activity to protect mice with LPS-induced septic shock by inhibiting the levels of IL-6 and TNF-α (Gao et al., 2018) and significantly ameliorate LPS-challenged acute kidney injury, to inhibit dimethylbenzene-induced mouse ear edema, and reduce LPS-induced sepsis in mice though the TLR4-MyD88–mediated NF and κB/MAPK signaling cascades (Yuan et al., 2019). Furthermore, DS has also been reported to dose-dependently attenuate endotoxin-induced HMGB1 release in macrophage and monocyte cultures, while HMGB1 has been a late mediator of lethal sepsis (Li et al., 2007). DG has been reported to effectively inhibit bacterial endotoxin–induced HMGB1 release *in vitro* and help mice defend against lethal endotoxemia and CLP-induced sepsis (Wang et al., 2006), and inhibit pro-inflammatory mediators IL-1β and TNF-α, thereby protecting LPS-induced endotoxic shock in rabbits (Shao et al., 2011b).

In the treatment of sepsis, XBJI provides an effective way for clinical treatment. However, the molecular mechanism in treating sepsis is still not clear. The mechanism of treatment of “bacteria, poison and inflammation” and effect of multi-ingredient, multi-target, and multi-pathway has still not been resolved. Therefore, it is necessary to elucidate the underlying material basis and molecular mechanism of XBJI in treating sepsis and get a clearer interpretation.

## 2 Materials and Methods

### 2.1 Pathogenetic Genes

We queried sepsis from GeneCards and DisGeNET with a total of 12 sepsis-related studies. The CUI IDs in DisGeNET were as follows: C0243026, C1719672, C0684256, C3164780, C0036686, C0152965, C0152964, C1141927, C0342959, C0036690, C0242966, and C0036685. The data obtained from these 12 studies were treated as pathogenetic genes of sepsis.

### 2.2 Construct Weighted Gene Regulatory Network of Xuebijing Injection

In order to construct comprehensive weight gene network of XBJI, the PPI data were derived from public web servers CMGRN and PTHGRN (Guan et al., 2014a, 2014b). Pathogenetic genes from DisGeNET (Piñero et al., 2017) were mapped to the PPI network and used in constructing the weighted gene regulatory network of XBJI. Cytoscape (Version 3.7.2) was utilized to visualize the network.

### 2.3 The Collection of Chemical Ingredients in Xuebijing Injection

The chemical ingredients of XBJI were collected from a published natural product data sources, TCMSP database (Ru et al., 2014). Besides, considering that chemical analysis plays an important role in the study of substance basis and mechanism of botanical drugs in the formulas, we also regarded some ingredients which had been detected in XBJI and reported in literatures as potential active ingredients.

### 2.4 Select Potential Active Ingredients of Xuebijing Injection Based on ADME Models

The potential active ingredients of XBJI in treating sepsis were selected from all chemical ingredients of XBJI based on ADME models, including Caco-2 permeability (Caco-2) and drug-likeness (DL) (Xu et al., 2012). The chemical ingredients with the criteria that Caco-2 > −0.4 and DL ≥ 0.18 were selected as potential active ingredients (Tao et al., 2013). According to literatures, some chemical ingredients which have high content and high biological activities were also selected as potential active ingredients of XBJI in treating sepsis.

### 2.5 Targets Prediction and the Construction of Ingredient–Targets Network

Targets of potential active ingredients of XBJI were predicted in three published databases, HitPick (Liu et al., 2013), similarity ensemble approach (Appearance Research Collaboration., 2014) (Tao et al., 2013), and SwissTargetPrediction (Gfeller et al., 2014). In order to evaluate the importance of targets, we conducted an ingredients–targets (I-T) network, investigated the node importance of ingredients and targets, and conducted KEGG and GO enrichment analysis of targets.

### 2.6 Construction of Quantitative Network Pharmacology Model

Node importance could reflect the effect of node in a complex network. Here, we designed a node importance calculation method to figure out the quantified influence of each node. The detail of the method can be described as follows:

$$Nim\left(s\right)=\sqrt[{2}]{\left(\overset{\;}{\underset{s\;\neq a\;\neq b\;\in V}{\sum }}\frac{\sigma_{ab\;(s)}}{\sigma_{ab}}\right)\;\times \;\left[\frac{\left|\;U\;\left(C_{\left(s\right)}\right)\right|}{\left|\;U\;\right|}\;\times \;\frac{\sum_{w\in C\left(s\right)}\left(\Delta_{C\left(s\right)}+1-dist_{\left(s,w\right)}\right)}{\max\;\left\{dist_{\left(s,w\right)}:w\in C\left(s\right)\right\}}\right]}$$

$$FRS=\;\left\{Nim_{i}\;\geq \;Mid\;\left\{\min\left(Nim\right)\;:\max\left(Nim\right)\right\},\;i\;\leq |U|\right\}$$

where, Nim represents the node of importance. $\sigma_{ab}$ represents the number of shortest paths between nodes a and b, $\sigma_{ab\left(s\right)}$ is the number of shortest paths passing through node s. C_(s)_ represents the genes which contain node s. U represents the collection of nodes within the network. |*U*| represents the number of nodes. $\Delta_{C\left(s\right)}$ is the maximum distance between gene C and the other genes passing through node s. dist_(s,w)_ represents the length of the shortest path between nodes s and w. The dist_(s,w)_ is equal to infinite if C_(s)_ ≠ C_(w)_, and it makes methods of this category inapplicable to networks with disconnected genes. Functional response space (FRS) represents the set of genes in the FRS; Med, Min, and Max represent median, minimum, and maximum, respectively. The genes in the FRS are defined as the key response proteins.

### 2.7 The Construction of Ingredients–Contribution–Proportion Model and the Selection of Critical Functional Ingredients Group

To optimize active ingredients and get the critical functional ingredients group (CFIG), which could be used to illustrate the molecular mechanism of XBJI in the therapy of sepsis, we constructed an ingredients–contribution–proportion (ICP) model, which could value the node importance in the network and contribute to the selection of the critical ingredients in representing the importance of the effect of all potential active ingredients.

We set m as the number of ingredients, I as the set of ingredients, I = [I_1_, I_2_, I_3_ … … I_m_], T as the set of targets of ingredients, T = [T1, T2, T3 … … T_m_]. After sorting the target number of ingredients in the descending order, we got the descending order set ST of the targets and the rearranged component set SI. SI was the new order of I after T in the descending order. At the same time, we set the set of optimized ingredients as Q and the set of optimized targets as W. CFIG was equal to Q. The ICP model can be described as:


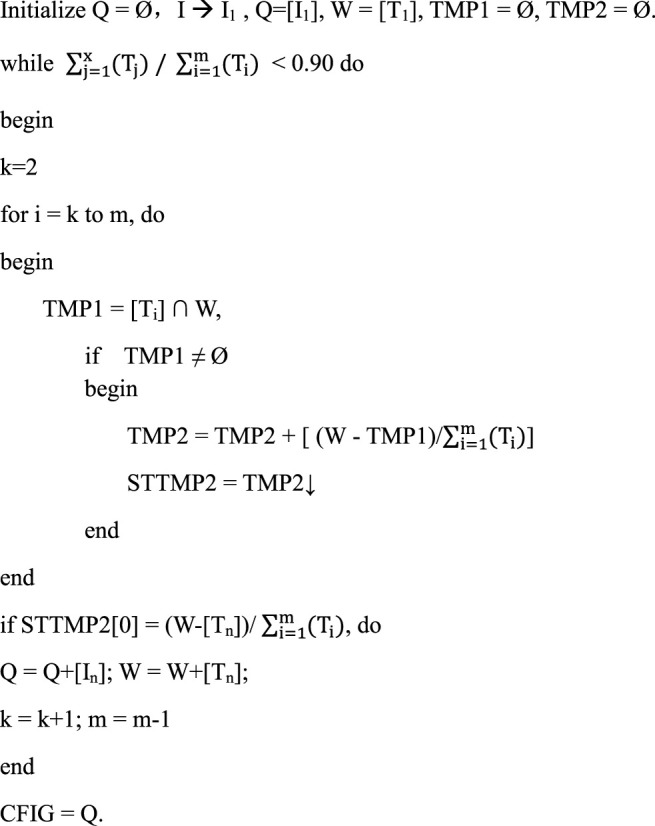


### 2.8 Gene Ontology and Pathway Analysis

For analyzing the main function of the targets, the clusterProfiler package and R Bioconductor (https://www.bioconductor.org/) were used to perform GO analysis (Yu et al., 2012) and KEGG pathway enrichment analysis (*p*-values = 0.05) (Draghici et al., 2007). The graphs creations and genes annotations were conducted with ggplot2 package and the Pathview (Luo et al., 2017), respectively.

### 2.9 Cell Verification

#### 2.9.1 The Selection of Three Ingredients

Three ingredients, apigenin, baicalein, and luteolin, were selected for *in vitro* experiments based on docking predictions and literatures. Here, docking predictions were conducted between the CFIG and proteins which were coded by the genes in the comprehensive pathways. The three-dimensional (3D) conformer of the CFIG was collected from ZINC (https://zinc.docking.org /) and PubChem (https://pubchem.ncbi.nlm.nih.gov /). The affinity method and pyMOL were conducted in docking prediction and graphs creation, respectively.

#### 2.9.2 Cell Culture and Treatment

Mouse macrophage RAW264.7 cells were obtained from the cell bank of the Chinese Academy of Sciences (Shanghai, China). The cells were cultured in complete Dulbecco’s Modified Eagle’s Medium (DMEM), including 8% fetal bovine serum (FBS), at 37°C in a constant temperature incubator with an atmosphere of 5% CO_2_. The culture medium was changed every 1–2 days; when RAW264.7 cells had reached approximately 80% confluence, they were blown down and passaged at a ratio of 1:2 or 1:3. When RAW264.7 cells reached 80% confluence, the cells were treated with apigenin (MOL000008, HPLC: 98.00%), baicalein (MOL002714, HPLC: 98.00%), and luteolin (MOL000006, HPLC: 98.00%) for 2 h, respectively. Then the cells were treated with LPS (Sigma-Aldrich, L4641) (1 μg/ml) for 24 h. FBS and DMEM were purchased from ThermoFisher Biochemical Products Co., Ltd (Beijing). Apigenin, baicalein, and luteolin were purchased from Jiangsu Yongjian Pharmaceutical Technology Co., Ltd (Jiangsu, China).

#### 2.9.3 Cell Viability Assay

The Cell Counting Kit-8 (CCK-8) was purchased from Dojindo Laboratories (Japan) and used to assess the cell viability. To test the cytotoxicity of the three ingredients, suspended RAW264.7 cells were placed onto 96-well plates at a density of 1×10^5^ cells/ml. After 24 h of incubation, cells had reached approximately 80% confluence; then treated with various concentrations of apigenin (1, 10, and 20 μM) (Park et al., 2020), baicalein (0.5, 1, 2, 4, and 8 μM) (Fan et al., 2013; Xiang et al., 2018; Zhang et al., 2021), and luteolin (1, 2, 4, and 8 μM) (Zhang et al., 2018; Zhou et al., 2020; Guo et al., 2021). Subsequently, 10 μl CCK-8 was added to each well. After 4 h of incubation, cell viability was determined by measuring the absorbance at 450 nm with a microplate reader (Infinite M200, TENAN, Switzerland).

#### 2.9.4 Assay the Content of NO

After incubation with apigenin, baicalein, and luteolin for 2 h, through the stimulation of LPS (1 ug/ml) for 24 h, the culture supernatant was collected and mixed with the total NO assay kit (Beyotime Institute of Biotechnology, China) for NO assay. The absorbance was measured at 540 nm with a microplate reader.

#### 2.9.5 Statistical Analysis

One-way analysis of variance for multiple comparisons and Student's t test for two groups' comparisons were utilized to analyze the significance of differences. The results were considered as statistically significant if the *p*-value was <0.05.

## 3 Results

**FIGURE 1:**
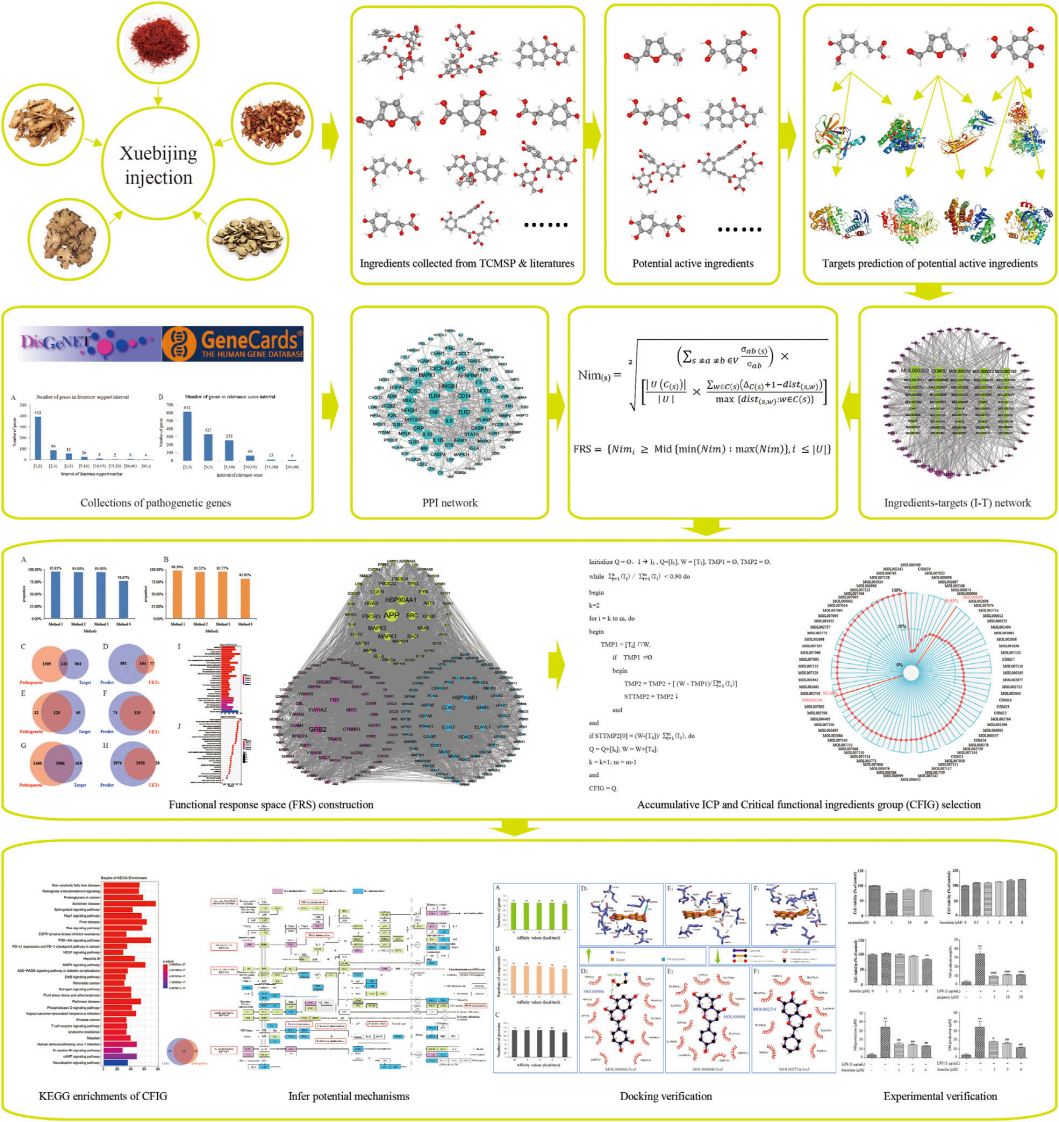
The work scheme of network pharmacology approach.

In this study, an integrative systematic pharmacology model was established to select the CFIG and clarify the therapeutic mechanism of XBJI in treating sepsis (Figure 1). Firstly, all ingredients of XBJI were collected from published databases and were used to select the potential active ingredients based on the proposed ADME-related models and literatures. Secondly, pathogenetic genes were collected from two published databases, GeneCards and DisGeNET, and were selected based on KEGG and GO enrichments. Thirdly, the weighted pathogenetic gene interactions and potential active ingredients targets network merged as a comprehensive network, which was input to a novel node influence calculation method to construct FRS for figuring out the key response proteins. Then, the reverse engineering–based ICP model was employed to select CFIG from the key response proteins. Fourthly, a compressive KEGG signaling pathway was used to uncover the hidden molecular mechanism of XBJI on sepsis. Finally, three ingredients of CFIG—apigenin, baicalein, and luteolin—were estimated by *in vitro* experiments with RAW264.7 cells.

### 3.1 Identification of Pathogenetic Genes

The process of sepsis is related to a series of complex changes in phenotype and gene expressions. These different phenotypic changes are accompanied by a number of alterations of genes expression. These genes may be labeled as pathogenetic genes both at diagnostic and intervention levels. The collection and analysis of pathogenetic genes are the foundational and critical ways in understanding the pathogenesis of sepsis and providing intervention strategies. For obtaining more comprehensive pathogenetic genes, we extracted pathogenetic genes from the proved sepsis-related literatures in DisGeNET and GeneCards databases (Supplementary Tables S1, S2).

**FIGURE 2:**
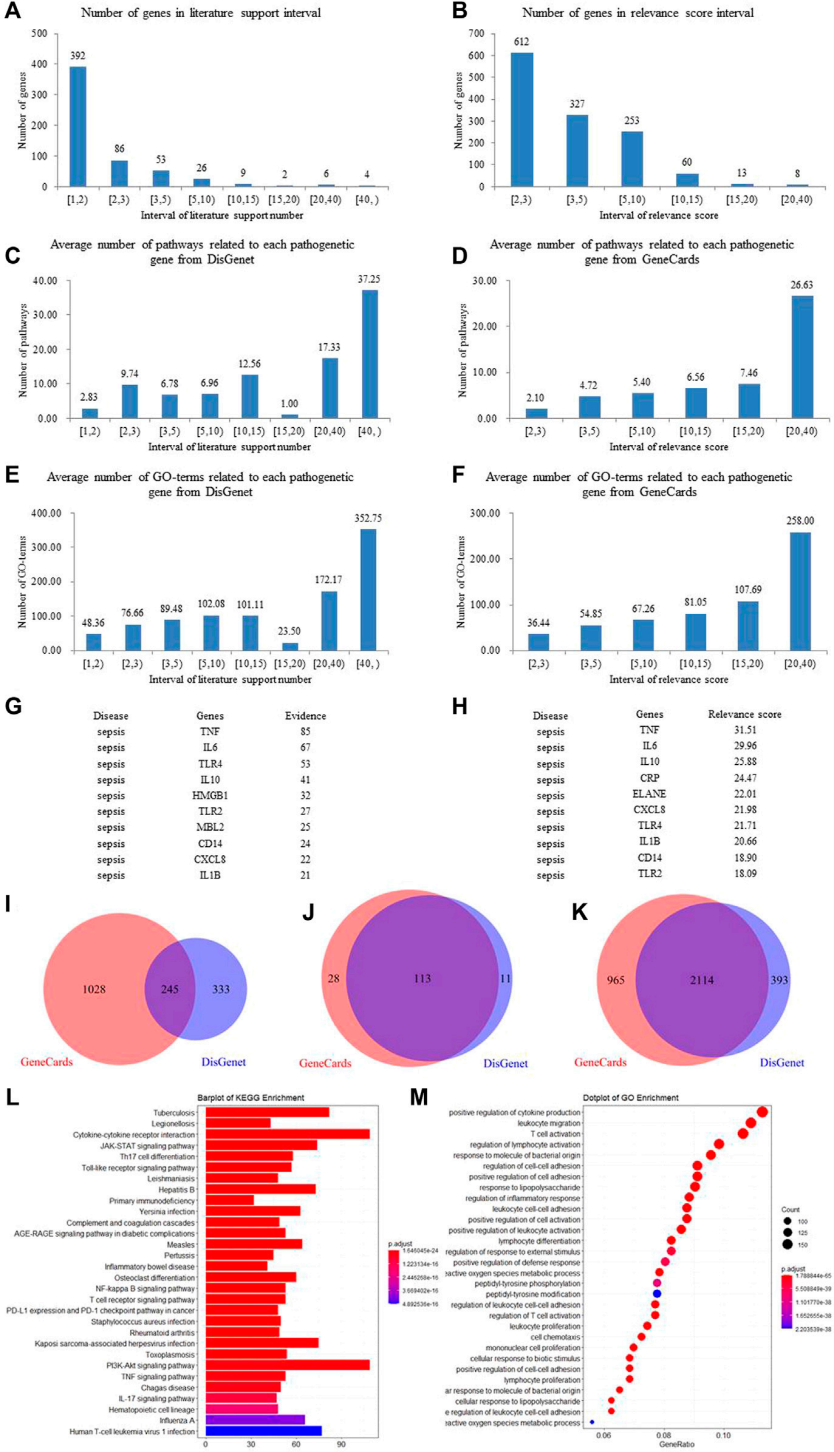
Study situations of pathogenetic genes. **(A,B)** Genes from DisGeNET and GeneCards, respectively. **(C,D)** KEGG enriched analysis of the genes from DisGeNET and GeneCards, respectively. **(E,F)** GO enriched analysis of the genes from DisGeNET and GeneCards, respectively. **(G,H)** The top 10 genes of DisGeNET and GeneCards valued on the numbers of supported literatures and relevance scores, respectively. **(I–K)** Interactions of genes, pathways, and GO-terms of the genes form DisGeNET and GeneCards. **(L,M)** KEGG and GO enrichments of pathogenetic genes.

Totally, 1,606 genes were obtained and were treated as pathogenetic genes, including 578 genes from DisGeNET and 1,273 genes from GeneCards. Among these 578 genes from DisGeNET, 186 genes were reported in more than 2 published reports (Figure 2A) and 21 genes were reported in more than 10 published reports. Among the 1,273 genes from GeneCards, the relevance scores of the 612 genes were equal or more than 2.00 but less than 3.00, and the relevance scores of the 81 genes were equal or more than 10.00 but less than 40.00. The top 10 genes with high numbers of supported reports in 578 genes from DisGeNET were TNF, IL-6, TLR4, IL-10, HMGB1, TLR2, MBL2, CD14, CXCL8, and IL-1β (Figure 2G). The top 10 genes with high relevance scores in 1,273 genes from GeneCards were TNF, IL-6, IL-10, CRP, ELANE, CXCL8, TLR4, IL-1B, CD14, and TLR2. TNF and IL-1β were typical pro-inflammatory factors and could reduce the body's immune function (Kamisoglu et al., 2015), while IL-6 and IL-10 were some anti-inflammatory cytokines (Chaudhry et al., 2013). Furthermore, many of these pathogenetic genes were reported to be related to sepsis closely. For example, TNF-α, a member of TNF, was usually detected in the blood of patients with sepsis (Fujishima, 2016) and was reported to induce NO production significantly (Ahn et al., 2012) and increase the death rate in LPS-induced sepsis mice (Li et al., 2020). IL-6 could mediate the inflammatory response of LPS-induced SIRS mice (Silva et al., 2019). TLR4 was a subtype of TLRs and could identify pathogenic bacteria in the innate immune system. It could activate IκB kinase (IKKs), cause the ubiquitination and degradation of IκB, activate NF-κB, and increase TNF-α expressions, thereby mediating hepatic inflammation, lung injury, and heart injury (Bieghs and Trautwein, 2014; Yin et al., 2018; Shen et al., 2019; Chang et al., 2021). HMGB1 was typically found in the nucleus of a variety of cells (immune, endothelial, epithelial cells, etc.). After the activation of TLR, HMGB1 could acetylate and trigger its translocation from the nucleus to the circulation and interact with a variety of target cell receptors (RAGE, TLR2, TLR4, etc.), stimulating the release of pro-inflammatory cyto-/chemokines and leading to inflammations and sepsis (Yang et al., 2015; Abdulmahdi et al., 2017; Andersson et al., 2018). CD14 was a receptor of LPS and was reported to mediate the occurrence and development of sepsis through the TLR4-NFκB pathway (Skirecki and Cavaillon, 2019).

To verify whether the genes supported by more literature in DisGeNET or by high score in GeneCards had a wide range of functions, we performed KEGG and GO enrichment to all pathogenetic genes and found that the number of most of the genes in the 578 genes from DisGeNET was directly proportional to the number of pathways and GO-terms. Genes supported by more literatures mostly had more pathways and GO-term correlations (Figure 2C,E). In addition, the relevance scores of all 1,273 genes from GeneCards were directly proportional to the number of pathways and GO-terms (Figure 2D,F). These results showed that the 1,606 genes from DisGeNET and GeneCards could represent the current occurrence and research of sepsis and could be used for pathogenetic genes in subsequent studies.

1,606 pathogenetic genes were enriched into 152 KEGG pathways and 3,446 GO-terms (Figure 2M). Among the 152 KEGG pathways, 8 pathways were reported to be associated with sepsis and were reported as cytokine–cytokine receptor interaction and some signaling pathways of JAK-STAT, TLR, NF-κB, T-cell receptor, PI3K-Akt, TNF, and IL-17. Among the 3,446 GO-terms, 8 GO-terms were reported to be associated with sepsis and were reported as positive regulation of cytokine production, leukocyte migration, T-cell activation, regulation of lymphocyte activation, regulation of peptide secretion, response to LPS, regulation of inflammatory response, and regulation of protein secretion.

The JAK/STAT signaling pathway had been proved to mediate the myocardial injury of septic rats with distinctly higher relative mRNA expression levels of JAK and STAT3 (Jin et al., 2020). Furthermore, JAK2/STAT3 signaling pathway had been demonstrated to induce the expression of miR-181b, which could downregulate sphingosine-1-phosphate receptor 1 (S1PR1) and decrease BBB cell adhesion, thereby promoting BBB impairment in rats with sepsis (Chen et al., 2020). TLR signaling was reported to be involved in cardiac redox signaling, calcium handling, energy metabolism, and affecting cardiac structure and gap junction proteins in sepsis mice (Lackner et al., 2020). The high expression of some proteins, such as TLR2, TLR3, and TLR4, have been demonstrated to play a crucial role in sepsis-induced acute injury of the lungs, kidneys, and intestine (Krivan et al., 2019; Kumar, 2020a). NF-κB and TNF-α were considered to be the links in regulating apoptosis (Zhao et al., 2015). Some studies showed that the cell damage caused by LPS was closely related to NF-κB, such as inflammatory apoptosis (Chand et al., 2018). The phosphorylation level of NF-κB was significantly increased in the lung tissues of sepsis mice (Wang et al., 2020). The activation of NF-κB would destroy intestinal barrier function during sepsis (Cao et al., 2021). Furthermore, the T-cell receptor signaling pathway was also demonstrated to be related to sepsis by network biology approach (Kim et al., 2021). The NFATC2 was upregulated in sepsis, which was also known as NFAT1, the first member of the nuclear factor of activated T cells (NFAT) family and had an important function in inducing gene transcription during an immune response (Qin et al., 2020). T-cell activation of both CD4+ and CD8+ T cells, as well as T-cell cytokine production, was suppressed acutely and persistently after burn injury, while sepsis could account for 47% of post burn mortality (Beckmann et al., 2020). Additionally, the activation of PI3K-Akt signaling pathway and the phosphorylation of Akt could attenuate apoptosis and improve survival in animal models of sepsis (Liu et al., 2019; Mizuta et al., 2020). The expression level of NF-κB and TNF-α was significantly higher in LPS-induced H9c2 cardiomyocytes, suggesting that the expression of NF-κB/TNF-α might be related to inflammatory response and apoptosis of H9c2 cells during sepsis (Zheng et al., 2020). The secretion of pro-inflammatory cytokine TNF-α was increased significantly in LPS-stimulated bone marrow–derived macrophages (BMDMs) (Liu et al., 2020). In the regulation of cytokine production, some cytokines had been proved to be beneficial for the control of sepsis, such as the anti-inflammatory cytokines of IL-1Ra, IL-4, IL-6, IL-10, IL-11, IL-13, IL-35, and TGF-β (Chaudhry et al., 2013). Studies showed that most of the differentially expressed proteins were mainly involved in leukocyte migration (Luo et al., 2021). The *trans*-endothelial leukocyte migration could be facilitated by miR-887-3p, thereby leading to an increase in leukocyte trafficking and dysregulation of inflammation and enhanced lung injury in sepsis-related acute respiratory distress syndrome (LeVine et al., 2000) (Goodwin et al., 2020). The latest study with a septic mouse model demonstrated that leukocyte transendothelial migration might be related to some downregulated lncRNAs (Huang et al., 2020). Septic patients were found to have a reduction in lymphocytes count (Coakley et al., 2019), which was because lymphocyte activation was negatively regulated by PD-1 ligands and PD-L1 (Kawamoto et al., 2019).

### 3.2 Construct Weighted Gene Regulatory Network of Sepsis

**FIGURE 3:**
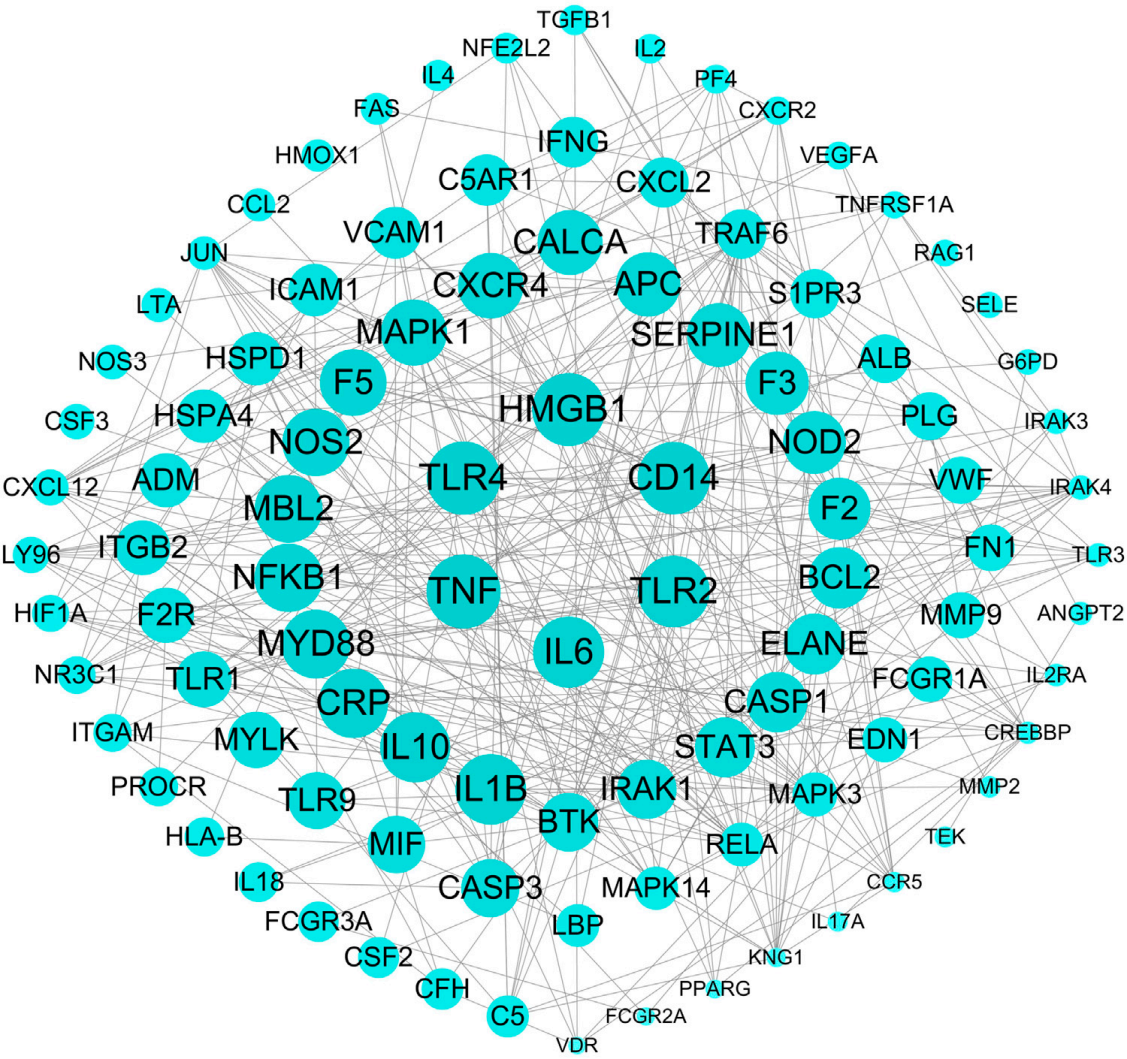
The top 100 weighted gene regulatory network of sepsis. The node size represents the weight of the genes in the network.

The weighted gene regulatory network can contribute to understand the pathogenetic genes and provide intervention strategies for sepsis. In order to construct weighted gene regulatory network, the base network which could show the relationship and interactions of genes was downloaded and combined from two published databases, including CMGRN and PTHGRN (Guan et al., 2014a, 2014b). 1,606 pathogenetic genes were constructed into a weighted gene regulatory network (PPI network) by mapping to the base network. The degree, relevance score, and number of literatures were multiplied to get the weight of the node in the regulatory network (Figure 3).

The weighted gene regulatory network contains 1,274 nodes and 22,560 edges (Figure 3). The number of edges represent the importance of the node in the network. Genes IL-6, TNF, TLR4, HMGB1, and CD14 were found to have higher weight in the top 100 weighted gene regulatory network. Some genes with high degrees have high supported literatures, such as genes TNF, TLR4, HMGB1, and TLR2 have scores of 37, 35, 46, and 25, respectively, larger than the average scores of 17.71 of all genes in the network, and also have the number of supported literatures of 85, 53, 32, and 27. Furthermore, some genes having a higher score in the PPI network also have a higher relevance score in the GeneCards database, such as genes TNF and ELANE have degrees of 37 and 23, respectively, higher than the average degree of 17.71 of all genes in the PPI network, and these also have high relevance scores of 31.51 and 22.01, respectively, in GeneCards. These genes with high degrees in the PPI network have been reported to participate in some signaling pathways associated with sepsis, such as the cytokine–cytokine receptor interaction (hsa04060), NF-κB signaling pathway, and MAPK3 signaling pathway. Among these pathways, some genes were reported to be associated with sepsis closely. For example, IL-6 could improve the stimulation and enhance the functions of T lymphocytes, B lymphocytes, and other cells involved in cell proliferation and differentiation, playing a role in anti-inflammation and regeneration. IL-10 was a powerful regulatory cytokine with anti-inflammatory effects and could inhibit the synthesis of a variety of cytokines, such as interferon c (IFN-c), TNF, and other pro-inflammatory cytokines. TNF-α was a pleiotropic cytokine which could trigger pro-inflammatory pathways by activating NF-κB to initiate the transcription of a variety of genes related to the production of inflammatory cytokines and could regulate the activity of inflammatory response promoters (AP-1) and other transcription factors through the MAPK3 signaling pathway. These results indicated that genes which were related to sepsis or to the activity of inflammation played important roles in the interaction in the PPI network and also that the PPI network could be used to further study FRS building.

### 3.3 Ingredients of Botanical Drugs in Xuebijing Injection

By a systematic search of ingredients from public databases of five botanical drugs in sepsis, including CS, CX, DS, DG, and HH, we obtained 728 ingredients (Supplementary Table S3). Meanwhile, we obtained 36 ingredients of XBJI according to literatures (Table 1).

**TABLE 1:** The information on the chemical analysis of the ingredients from the literature in five botanical drugs.

ID	Ingredient	Method	Concentration	Ref
COM1	4′,5,6,7-Tetrahydroxyflavanone 6,7-di-O-b-D-glucopyranoside	HPLC-ESI-MS	—	Huang et al. (2011)
COM2	5-Hydroxymethylfurfural	UHPLC	10.38 μg/ml	Zuo et al. (2017)
COM3	6-Hydroxykaemferol 3,6,7-tri-O-b-D-glucopyranoside	HPLC-ESI-MS	—	Huang et al. (2011)
COM4	6-Hydroxykaemferol 3,6-di-O-b-Dglucopyranosyl-7-O-b-Dglucuronopyranoside	HPLC-ESI-MS	—	Huang et al. (2011)
COM5	Anhydrosafflor yellow B	HPLC-ESI-MS	—	Huang et al. (2011)
COM6	Benzoylpaeoniflorin	UHPLC	39.82 μg/ml	Zuo et al. (2017)
COM7	Butylidenephthalide	UHPLC	0.02 μg/ml	Zuo et al. (2017)
COM8	Catechinic acid	UHPLC	5.76 μg/ml	Zuo et al. (2017)
COM9	Chlorogenic Acid	UHPLC	3.92 μg/ml	Zuo et al. (2017)
COM10	Ethyl ferulate	UHPLC	0.19 μg/ml	Zuo et al. (2017)
COM11	Gallic acid	UHPLC	6.60 μg/ml	Zuo et al. (2017)
COM12	Galuteolin	UHPLC	1.10 μg/ml	Zuo et al. (2017)
COM13	Hydroxysafflor yellow A	UHPLC	479.45 μg/ml	Zuo et al. (2017)
COM14	Hyperoside	UHPLC	0.63 μg/ml	Zuo et al. (2017)
COM15	Naringenin	UHPLC	0.35 μg/ml	Zuo et al. (2017)
COM16	Protocatechuic aldehyde	HPLC	7.28 mg/L	Ji et al. (2010)
COM17	Rosmarinic acid	UHPLC	5.07 μg/ml	Zuo et al. (2017)
COM18	Safflor yellow A	HPLC	48.79 mg/L	Ji et al. (2010)
COM19	Senkyunolide H	HPLC	19.48 mg/L	Ji et al. (2010)
COM20	Senkyunolide I	HPLC	53.89 mg/L	Ji et al. (2010)
COM21	Sodium Danshensu	UHPLC	0.49 μg/ml	Zuo et al. (2017)
COM22	Tanshinone IIA	UHPLC	0.10 μg/ml	Zuo et al. (2017)
COM23	Uridine	HPLC	38.61 mg/L	Ji et al. (2010)
MOL000105	Protocatechuic acid	UHPLC	4.81 μg/ml	Zuo et al. (2017)
MOL000223	Caffeic acid	UHPLC	5.25 μg/ml	Zuo et al. (2017)
MOL000389	Ferulic acid	UHPLC	35.12 μg/ml	Zuo et al. (2017)
MOL000415	Rutin	UHPLC	2.27 μg/ml	Zuo et al. (2017)
MOL000874	Paeonol	UHPLC	0.03 μg/ml	Zuo et al. (2017)
MOL001924	Paeoniflorin	HPLC	1963.86 mg/L	Ji et al. (2010)
MOL001932	Galloylpaeoniflorin	HPLC-ESI-MS	—	Huang et al. (2011)
MOL002687	Guanosine	HPLC	27.15 mg/L	Ji et al. (2010)
MOL007004	Albiflorin	UHPLC	26.24 μg/ml	Zuo et al. (2017)
MOL007006	Oxypaeoniflorin	UHPLC	40.54 μg/ml	Zuo et al. (2017)
MOL007074	Salvianolic acid B	UHPLC	2.14 μg/ml	Zuo et al. (2017)
MOL007134	Danshensu	HPLC	48.49 mg/L	Ji et al. (2010)
MOL007136	Salvianolic acid A	UHPLC	0.04 μg/ml	Zuo et al. (2017)

### 3.4 Select Potential Active Ingredients

**FIGURE 4:**
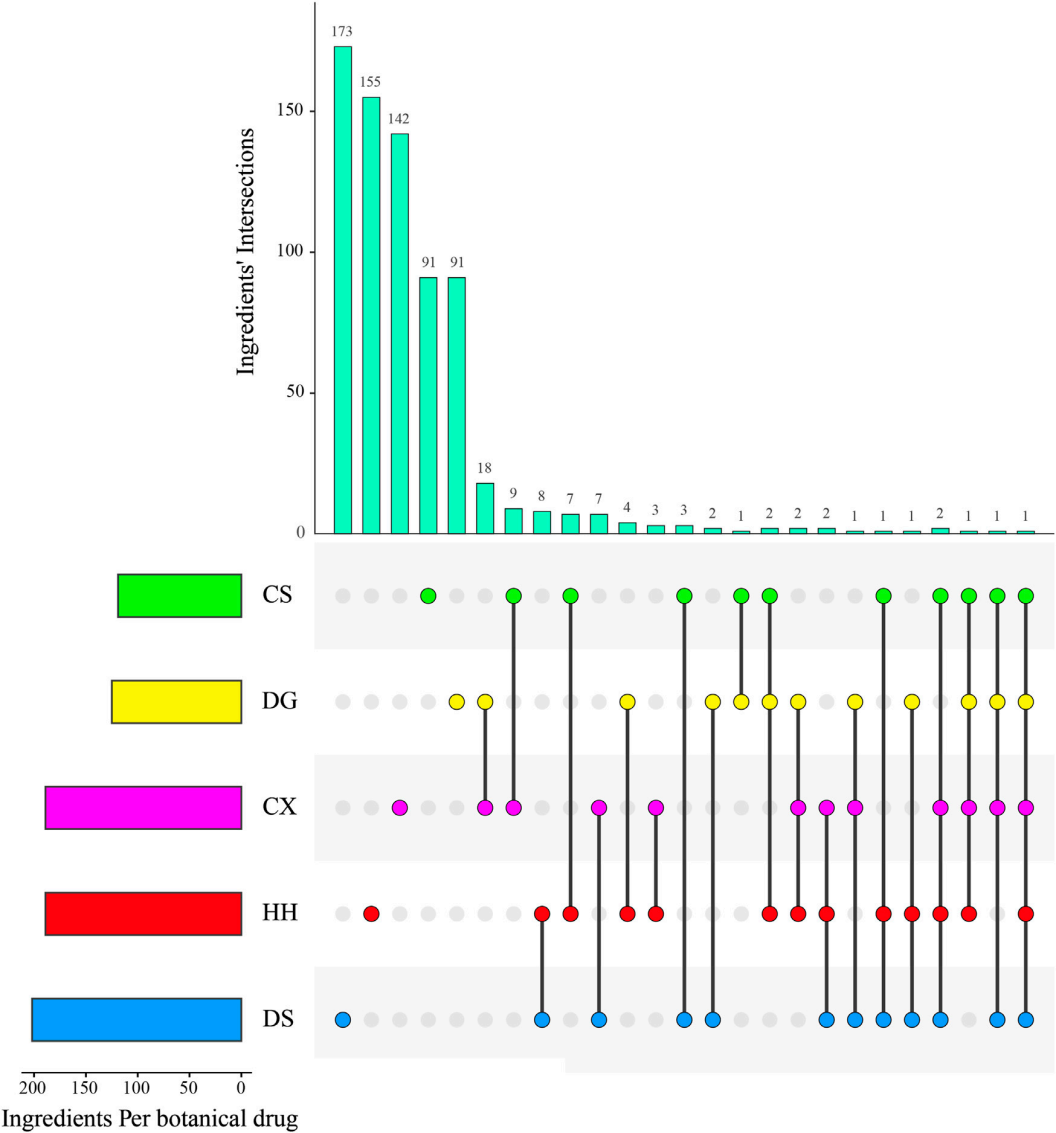
Ingredients' intersections of botanical drugs in XBJI.

We obtained 205 potential active ingredients from these 728 ingredient base on two ADME-related models, including Caco-2 and DL (Figure 4). Considering that chemical analysis plays important roles in the study of substances basis and mechanism of botanical drugs in the formulas, we regarded the 36 ingredients collected from literatures as potential active ingredients. As a result, we collected 241 potential active ingredients of XBJI (Table 2, Supplementary Table S4).

**TABLE 2:** Statistics on the number of XBJI ingredients collected in the database and from literatures.

Botanical drugs	Ingredients from TCMSP	Selected ingredients from TCMSP	Selected ingredients of XBJI from literatures
CS	119	31	36
CX	189	18	
DS	202	114	
DG	125	8	
HH	189	50	
Total	**728**	**205**	**36**

### 3.5 Shared Ingredients of Botanical Drugs in Xuebijing Injection

A total of one ingredient, palmitic acid (MOL000069), is contained in five botanical drugs. Four ingredients were contained in four botanical drugs, and they are EIC (MOL000131), sitogluside (MOL000357), moslene (MOL000202), and oleic acid (MOL000675). Nine ingredients were contained in three botanical drugs, and these are baicalin (MOL002776), beta-sitosterol (MOL000358), stigmasterol (MOL000449), nonanal (MOL000116), cymol (MOL000117), adenine (MOL001788), caffeic acid (MOL000223), stearic acid (MOL000860), and succinic acid (MOL000346). In addition, 62 ingredients were contained in two botanical drugs (Figure 4, Supplementary Table S5). Among them, palmitic acid was reported to increase serum levels of TNF-α, IL-6, IL-10 and to activate TLR4 and downstream inflammatory responses by binding to TLR4 accessory protein MD2 directly, thereby regulating the TLR4 signaling pathway, MAPKs/NF-κB signal pathway, and the level of pro-inflammatory molecules (Wang et al., 2017; Panpetch et al., 2020). Oleic acid was reported to be higher in septic patients (Mecatti et al., 2018) and to decrease plasma-free FA concentration and increase the levels of CPT1A, UCP2, and AMPK, thereby decreasing the levels of reactive oxygen species (ROS) in septic mice (Gonçalves-de-Albuquerque et al., 2016). Baicalin could significantly reduce IL-1β in the sera of bacterial infected mice and could inhibit NLRP3 inflammasome activation through augmenting PKA signaling, thereby improving mouse survival in bacterial sepsis (Li et al., 2017) and could reduce the level of LPS, TNF-α, and IL-6 in the blood of sepsis mice (Chen et al., 2016). Besides CS, DG, and HH, beta-sitosterol was also found to exist in *Hechtia glomerata* Zucc., which was used as a source in ethnomedicine to treat various diseases derived from bacterial infections, such as sepsis, bronchitis, and laryngitis (Stefani et al., 2019). Beta-sitosterol was predicted to mediate NF-κB signaling pathway (Kasirzadeh et al., 2021) and was found to significantly reduce the pro-inflammatory cytokines in the sera of cecal ligation and puncture-induced septic rats, such as TNF-α and NF-κB mRNA expressions. In addition, stigmasterol was proved to decrease the levels of cyclooxygenase-2 (Cox-2) and NF-κB (p65) in the brain of rats, thereby attenuating inflammation and improving antioxidant defenses (Liang et al., 2020). Caffeic acid could reduce pro-inflammatory cytokines IL-1β, IL-6, and TNF-α and attenuate iNOS and COX-2 in LPS-stimulated macrophages (Chen et al., 2019).

### 3.6 Specific Ingredients of Botanical Drugs in Xuebijing Injection

Except the shared ingredients, most of the botanical drugs have its specific ingredients (Supplementary Table S5). For example, the number of unique ingredients of CS, CX, DS, DG, and HH are 91, 142, 173, 91, and 155, respectively (Figure 4).

According literatures, these unique ingredients in different botanical drugs have special therapeutic effects. For example, paeonol (MOL000874), paeoniflorin (MOL001924), galloylpaeoniflorin (MOL001932), albiflorin (MOL007004), and oxypaeoniflorin (MOL007006) are the specific ingredients of CS. Among them, paeonol was reported to promote phagocytosis of macrophages through confining HMGB1 to the nucleus, thereby promoting the immune response during sepsis (Miao et al., 2020) and attenuate the inflammation mediated by HMGB1 and IKK-β by upregulating miR-339-5p expression, thereby protecting the kidneys and improving the survival rate of sepsis mice (Mei et al., 2019). Paeoniflorin was a critical ingredient of monoterpene glycoside in CS and XBJI (Wang et al., 2020). It was reported to reduce inflammation, prevent MODS, and protect organ functions by reducing the plasma levels of aspartate aminotransferase (AST), creatine kinase-MB, and soluble triggering receptor expressed on myeloid cells-1 (Liu et al., 2016). Furthermore, it has been reported to improve myocardial function in septic mice by attenuating LPS-induced myocardial inflammatory cytokines production, such as TNF-α, IFN-γ, IL-1β, IL-6, IL-12, and MCP-1 (Zhai and Guo, 2016). Galloylpaeoniflorin had the same structural characteristics of paeoniflorin, which had significant NF-κB inhibitory effects (Lu et al., 2019). Oxypaeoniflorin was proved to be an important ingredient in XBJI with a significant effect in inhibiting NF-κB, IL-6, and IL-8 in LPS-induced human monocyte THP-1 cells (Jiang et al., 2013). Protocatechuic acid (MOL000105) exists in CX and was proved to be an important ingredient in XBJI (Li et al., 2016; Li et al., 2019). Its derivative, protocatechuic acid isopropyl ester, had been proved to protect murine model against sepsis by inhibiting TNF-α production, NO production, and the augmentation of IL-6 and IL-10 (Yan et al., 2004). Salvianolic acid B (MOL007074) is a water-soluble ingredient in DS and had been detected to be an important ingredient in XBJI (Huang et al., 2011; Li et al., 2016; Li et al., 2019; Xing et al., 2020). It was demonstrated to exert an antiviral effect (Chung et al., 2015) and was supposed to be useful in treating COVID-19 (Xing et al., 2020). Danshensu (MOL007134), an ingredient in DS, had been detected in XBJI and was reported to inhibit the overexpression of NF-κB in human embryonic kidney 293 (HEK 293) cells which was significantly stimulated by TNF-α (Jiang et al., 2013). Ferulic acid (CIS) (MOL000389), an ingredient in DG, had been reported to play a protective role on sepsis-induced oxidative damage by significantly decreasing malondialdehyde levels (Salomão et al., 2008), significantly increasing glutathione (GSH) levels and superoxide dismutase (Deng et al., 2013), and glutathione peroxidase (GSH-Px) enzyme activities in Wistar albino rats (Bacanlı et al., 2014). Furthermore, it was reported to positively modulate the inflammatory response to septic liver injury through the GSK-3β/NF-κB/CREB pathway, including decreasing the activities of MPO, AST, and alanine aminotrasferase (ALT), and including decreasing the levels of some pro-inflammatory factors, IL-1β, IL-6, IL-10, IL-12, and TNF-α (Cao et al., 2021).

### 3.7 Construction of I-T Network

**FIGURE 5:**
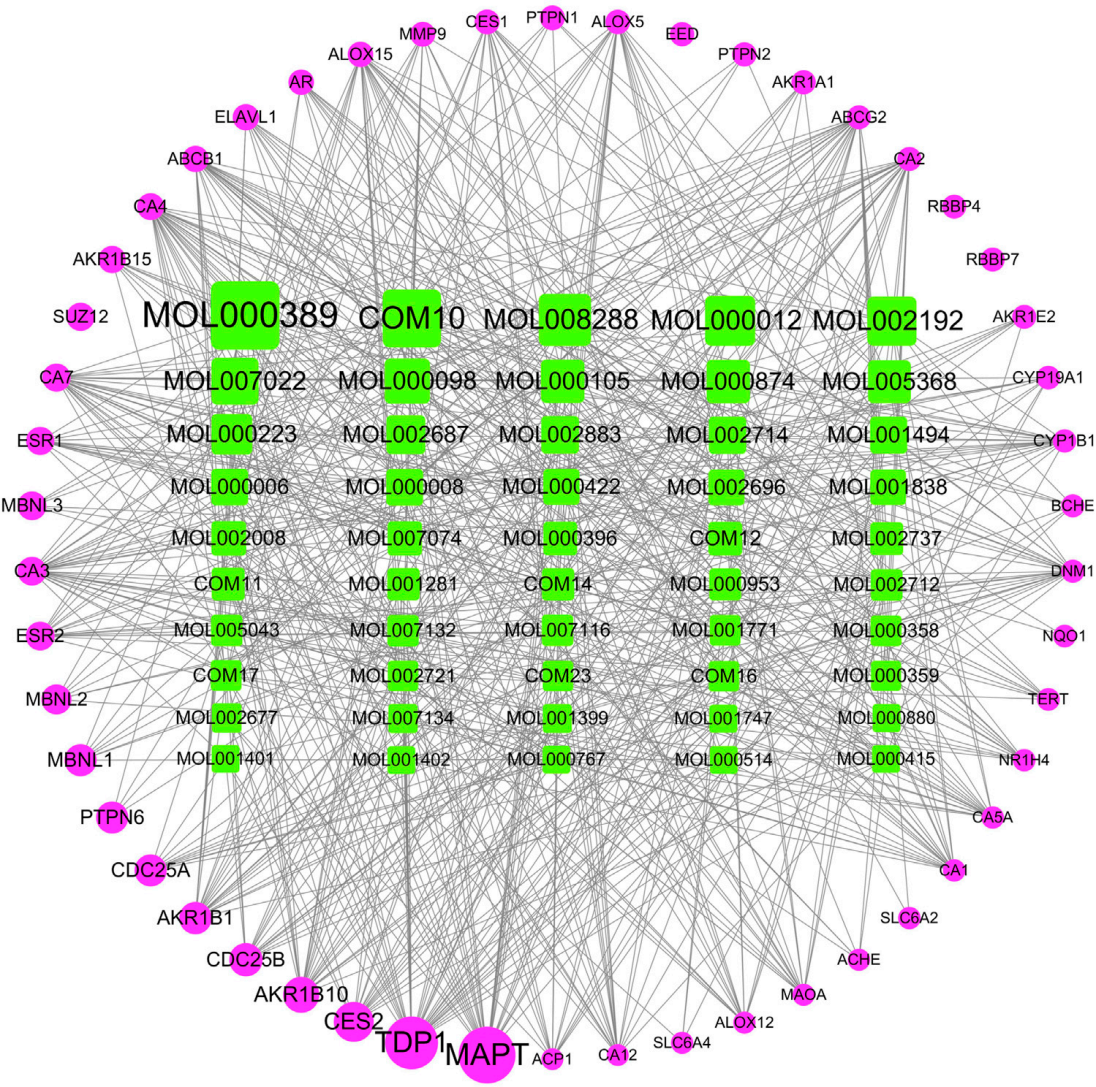
I-T network. The top 50 ingredients and top 50 targets valued by the degree in the I-T network are shown in Figure 5. Green represents potentially active ingredients, pink represents targets; node size represents the importance of the genes in the network.

For exploring the therapeutic mechanism of XBJI in treating sepsis, 241 potential active ingredients and 1,105 targets were used in constructing the I-T network (Supplementary Table S6). The top 50 ingredients and top 50 targets valued by the degree in the I-T network are shown in Figure 5. Some of these potential active ingredients are related multiple targets, resulting in that 8,598 I-T associations between all the potential active ingredients and targets in the I-T network. The average number of targets of per ingredient is 35.68, indicating that there are multi-ingredients and multi-targets characteristics of XBJI in treating sepsis. Among these potential active ingredients, ferulic acid (CIS) (MOL000389) has the highest number of targets as 185, followed by ingredients ethyl ferulate (COM10) and coniferyl ferulate (MOL008288) with the number of targets as 151 and 131, respectively. Among these ingredients, ferulic acid (CIS) had been proved to decrease sepsis-induced oxidative damage (Bacanlı et al., 2014) and protect the liver during sepsis through the GSK-3β/NF-κB/CREB pathway (Cao et al., 2021). Ethyl ferulate was proved to inhibit the inflammatory responses in RAW264.7 cells and acute lung injury of mice by activating the Nrf2/HO-1 pathway and inhibiting the NF-κB pathway (Wang et al., 2021). Coniferyl ferulate was proved to inhibit the activation of NMDAR-CaMKII-MAPKs and mitochondrial apoptotic pathways, including inhibiting the generation of ROS, decreasing the activity of SOD, thereby significantly attenuating the decrease of cell viability (Gong et al., 2020). These results demonstrated that the potential active ingredients played important roles in treating sepsis.

### 3.8 Key Response Proteins Selection and Validation From Functional Response Space

Here, we constructed a disease–targets network based on the weighted gene regulatory network and I-T network. This network contains 2,110 nodes and 58,258 edges (Supplementary Table S7).

Node importance is an important topological property and can be used to evaluate the influence of nodes in the network. The nodes whose node importance are larger than the average node importance of all nodes are treated as critical roles and hub nodes in the network (Liu et al., 2016). Here, we designed a novel node importance calculation method to figure out the importance and the influence of genes. According to this rule, these nodes with higher importance scores than average importance score were kept and integrated with their edges to form FRS.

In order to evaluate the effectiveness of the FRS, we defined the intersection of pathogenetic genes and XBJI targets as the un-optimized effective targets (UETs) of XBJI and defined the genes which were included in the FRS as the key response proteins. Then, we evaluated the key response proteins with three evaluation indicators: 1) the proportion of key response proteins in the number of UETs, 2) the proportion of key response proteins in the number of UETs enriched pathways, and 3) the proportion of the key response proteins in the number of UETs enriched GO-terms.

**FIGURE 6:**
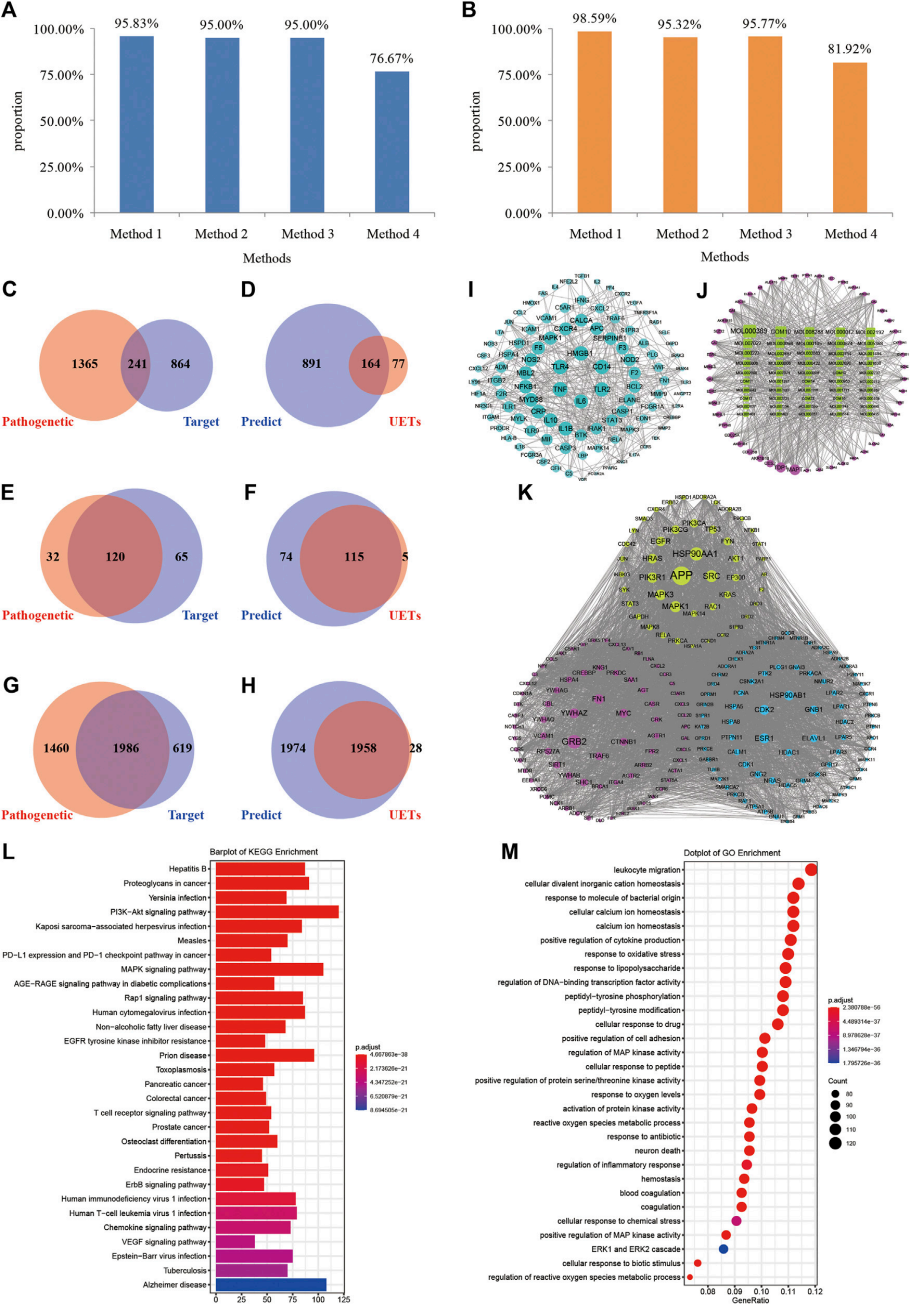
Construction and validation of FRS. **(A–B)** Method 1, method 2, method 3, and method 4 represent the novel node importance calculation methods we proposed, Degree, Closeness Centrality, and Clustering Coefficient, respectively. **(C)** The number of pathogenetic genes and targets; **(D)** The number of the genes in FRS and UETs; **(E)** The number of signaling pathways of pathogenetic genes and targets, respectively; **(F)** The number of signaling pathways of the genes in FRS and UETs, respectively; **(G)** The number of GO-terms of pathogenetic genes and targets, respectively; **(H)** The number of GO-terms of the genes in FRS and UETs, respectively; **(I)** PPI network; **(J)** I-T network; **(K)** The top 200 genes in FRS are shown in the figure and include three categories of targets. **(L)** KEGG enrichment analyses of the genes in FRS; **(M)** GO-terms of the genes in FRS. Red nodes represent disease-specific targets, green nodes represent essential common targets, and blue nodes represent ingredient-specific targets.

Before the optimization, the number of XBJI targets and pathogenetic genes of sepsis were 1,105 and 1,606, respectively (Figure 6C); the numbers of targets and pathogenetic genes enriched pathways were 185 and 152 (*p* < 0.05), respectively; while, the numbers of targets and pathogenetic genes enriched GO-terms were 2,605 and 3,446, respectively (*p* < 0.05). We used four methods in the optimization of targets and pathogenetic genes. Our proposed node importance calculation method was better than three other traditional methods: Degree, Closeness Centrality, and Clustering Coefficient (Figure 6D). Based on our method, 1,055 nodes were filtrated as important nodes and were defined as the key response proteins. The key response proteins and their interactions were used to construct the FRS. There were three subtypes of key response proteins in the FRS (Figure 6K). The first subtype represents essential common targets, which directly linked pathogenetic genes and ingredients' targets. The second subtype represents disease-specific targets. The third subtype represents ingredient-specific targets.

The number of key response proteins enriched pathways and GO-terms were 189 and 3,932, respectively (Figures 6L,M). The enriched pathways accounted for 95.83% of UETs enriched pathways (Figure 6F). The GO-terms accounted for 98.59% of UETs enriched GO-terms (Figure 6H). These high coverages confirmed the accuracy and reliability of our method in constructing the FRS.

Furthermore, in order to validate whether the key response proteins stored in the FRS could represent the importance of pathogenetic genes of sepsis, KEGG, and GO enrichments, analysis was conducted for the key response proteins.

KEGG enrichments showed that the key response proteins could be involved in 189 pathways, and mainly involved in some signaling pathways of PI3K-Akt (hsa04151), MAPK (hsa04010), Rap1 (hsa04015), human cytomegalovirus infection (hsa05163), and T-cell receptor (hsa04660), etc. Many of these pathways had been reported to have close association with sepsis. For example, the activation of PI3K-Akt signaling pathway and the phosphorylation of Akt could attenuate apoptosis and improve survival in animal models of sepsis (Liu et al., 2019; Mizuta et al., 2020). The activation of the MAPK signaling pathway was reported to promote sepsis-induced myocardial injury by overexpressing the level of angiotensin II type 1 receptor (AT1R). The signaling cascades of TLR4-MyD88-NF-κB/MAPK could be effected by DS, thereby significantly ameliorating LPS-challenged acute kidney injury, inhibiting dimethylbenzene-induced mouse ear edema, and rescuing LPS-induced sepsis in mice (Yuan et al., 2019). The activation of the PI3kinase/RAP1 signaling pathway could lead to the activation of integrin receptor GPIIbIIIa and the release of dense granules, contributing to the thrombotic complications of sepsis (Keane et al., 2010). Furthermore, the T-cell receptor signaling pathway was reported to release some activated T cells and nuclear factors, CD4^+^, CD8^+^, and NFATC2, to take the immune response in sepsis (Beckmann et al., 2020; Kim et al., 2021).

GO enrichments showed that the key response proteins could be enriched into 3,932 GO-terms (Figure 6H), including leukocyte migration (GO:0050900), response to oxidative stress (GO:0006979), response to LPS (GO:0032496), cellular response to drugs (GO:0035690), neutrophil activation (GO:0042119), etc. Many of these GO-terms had been reported to have a close association with sepsis. For example, some of the differentially expressed proteins in plasma of septic patients acted on leukocyte transendothelial migration (Luo et al., 2021), and leukocytes from septic patients could reverse glucose metabolism to oxidative phosphorylation from glycolysis and display multiple energy-metabolizing defects (Cheng et al., 2016). Some proteins and receptors had been proved to be related with oxidative stress, oxygen species metabolic process, etc., during sepsis. For example, heat shock protein 70 in the intracellular environment was reported to have chaperone activity, correcting damaged proteins and modulating inflammatory response (Sulzbacher et al., 2020); P2X7 receptor blockers were reported to limit oxidative damage and inflammations in sepsis (Larrouyet-Sarto et al., 2020); the overexpression of protein kininogen-1 (KNG1) could strengthen inflammation and oxidation in sepsis-induced acute lung injury (Hu et al., 2020). LPS was reported to effect diminishing the intracellular alkalization and change neutrophil size induced by platelet-activating factor (PAF), which was an important mediator of the systemic inflammatory response (Hug et al., 2021), and have an effect on the phagocytosis and reactive oxygen species (ROS) production of neutrophils (Messerer et al., 2020). The depolarization of membrane potential of neutrophils could be significantly impaired by PAF, thereby inducing neutrophil dysfunctions during sepsis (Hug et al., 2021). During the late stage of sepsis, CD4^+^ and CD8^+^ and other T cells become apoptotic and responsible for the development of lymphopenia and immunosuppression, thereby exacerbating the deterioration of the sepsis condition (Rimmelé et al., 2016; Kumar, 2018).

### 3.9 Critical Functional Ingredients Group Selection and Validation

**FIGURE 7:**
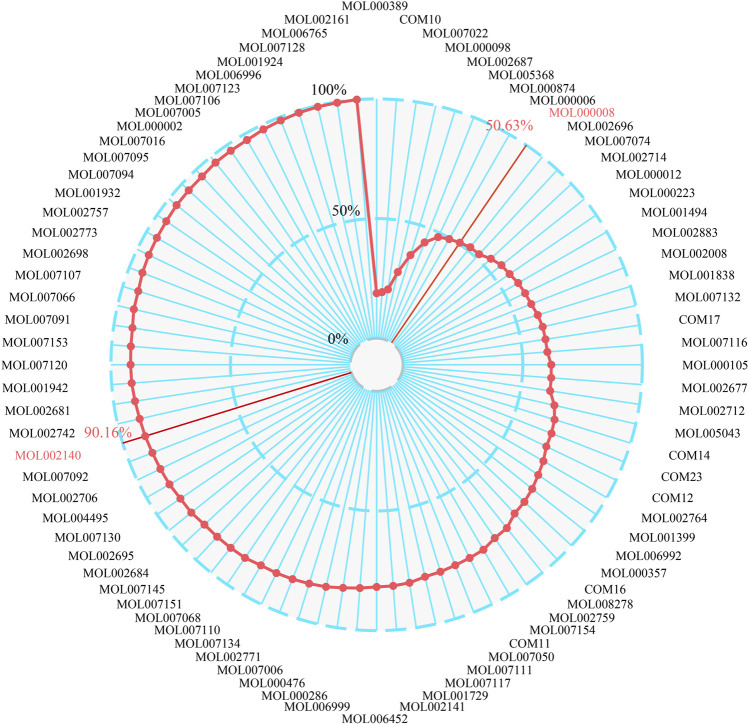
The ICP and accumulative ICP of active ingredients in XBJI.

The CFIG of XBJI in treating sepsis was united with the effective ingredients which were established from the FRS with the ICP model. According to the contribution accumulation score ranking, targets of the top nine ingredients including ferulic acid (MOL000389), ethyl ferulate (COM10), evofolin B (MOL007022), quercetin (MOL000098), guanosine (MOL002687), methyl tricosanoate (MOL005368), paeonol (MOL000874), luteolin (MOL000006), and apigenin (MOL000008) could cover 50.63% of the key response proteins. For further analysis, targets of the top 60 ingredients could cover 90.16% of the key response proteins, and these 60 ingredients were selected as the CFIG (Figure 7; Table 3, Supplementary Figure S1). The high targets coverage of key response proteins of 90.16% proved that the CFIG of XBJI played the leading role in the therapy of sepsis.

**TABLE 3:** Information of the ingredients in CFIG of XBJI.

ID	Ingredient name	Caco-2	DL	ID	Ingredient name	Caco-2	DL
MOL000006	Luteolin	0.19	0.25	MOL004495	Tigogenin	0.87	0.81
MOL000008	Apigenin	0.43	0.21	MOL005043	Campest-5-en-3beta-ol	1.32	0.71
MOL000012	Arachic acid	1.18	0.19	MOL005368	Methyl tricosanoate	1.43	0.33
MOL000098	Quercetin	0.05	0.28	MOL006452	1,5-Dihydroxy-3-methylanthraquinone	0.56	0.21
MOL000105	Protocatechuic acid	0.10	0.04	MOL006992	(2R,3R)-4-Methoxyl-distylin	0.17	0.30
MOL000223	Caffeic acid	0.21	0.05	MOL006999	Stigmast-7-en-3-ol	1.32	0.75
MOL000286	β-Amyrin acetate	1.42	0.74	MOL007006	Oxypaeoniflorin	−1.91	0.78
MOL000357	Sitogluside	−0.14	0.62	MOL007022	Evofolin B	0	0.22
MOL000389	Ferulic acid (CIS)	0.53	0.06	MOL007050	2-(4-hydroxy-3-methoxyphenyl)-5-(3-hydroxypropyl)-7-methoxy-3-benzofurancarboxaldehyde	0.35	0.40
MOL000476	Physcion	0.52	0.27	MOL007068	Przewaquinone B	0.39	0.41
MOL000874	Paeonol	0.93	0.04	MOL007074	Salvianolic acid B	−1.67	0.41
MOL001399	TWT	1.85	0.18	MOL007092	Dan-shexinkum c	0.75	0.21
MOL001494	Mandenol	1.46	0.19	MOL007110	Isotanshinone IIb	0.38	0.45
MOL001729	Crysophanol	0.62	0.21	MOL007111	Isotanshinone II	1.03	0.40
MOL001838	Dipalmitin	0.39	0.44	MOL007116	Methylrosmarinate	0	0.37
MOL002008	Myricetin	−0.15	0.31	MOL007117	Methyltanshinonate	0.56	0.55
MOL002140	Perlolyrine	0.88	0.27	MOL007130	Prolithospermic acid	0.10	0.31
MOL002141	PLO	0.69	0.43	MOL007132	(2R)-3-(3,4-dihydroxyphenyl)-2-[(Z)-3-(3,4-dihydroxyphenyl)acryloyl]oxy-propionic acid	−0.33	0.35
MOL002677	L-1,2-Dipalmitin	0.38	0.49	MOL007134	Danshensu	−0.27	0.06
MOL002684	gamma-Tocotrienol	1.55	0.53	MOL007145	Salviolone	1.04	0.24
MOL002687	Guanosine	−1.21	0.21	MOL007151	Tanshindiol B	0.05	0.45
MOL002695	Lignan	0.42	0.65	MOL007154	Tanshinone IIa	1.05	0.40
MOL002696	Lirioresinol A	0.41	0.67	MOL008278	4-chloro-N-[1-methyl-5-[[1-methyl-5-[[1-methyl-5-(2-morpholinoethylcarbamoyl)pyrrol-3-yl]carbamoyl]pyrrol-3-yl]carbamoyl]pyrrol-3-yl]-5-[2-(2-pyridyl)ethylamino]isothiazole-3-carboxamide	−0.21	0.31
MOL002706	Phytoene	2.22	0.5	COM10	Ethyl ferulate	—	—
MOL002712	6-Hydroxykaempferol	0.16	0.27	COM11	Gallic acid	—	—
MOL002714	Baicalein	0.63	0.21	COM12	Galuteolin	—	—
MOL002759	Glyceryl pps	0.45	0.35	COM14	Hyperoside	—	—
MOL002764	MEGxp0_000365	−0.12	0.53	COM16	Protocatechuic aldehyde	—	—
MOL002771	VIV	1.70	0.55	COM17	Rosmarinic acid	—	—
MOL002883	Ethyl oleate (NF)	1.40	0.19	COM23	Uridine	—	—

Most of the CFIG had been proved to state and be high enough in XBJI or have a close association with sepsis and anti-inflammatory responses. For example, three ingredients in the CFIG, namely, ferulic acid, guanosine, and paeonol, had been reported to state and be high enough, with concentrations of 35.12, 27.15, and 0.04 μg/ml, respectively, in XBJI (Ji et al., 2010; Zuo et al., 2017). Ferulic acid is a characteristic ingredient in DG and has the highest number of targets as 185 in our I-T network. It had been proved to protect Wistar albino rats from sepsis-induced oxidative damage (Bacanlı et al., 2014) and positively modulate the inflammatory response to septic liver injury through the GSK-3β/NF-κB/CREB pathway (Cao et al., 2021). Ethyl ferulate has the secondary number of targets as 151, following ferulic acid in the I-T network and has been proved to inhibit the inflammatory responses in RAW264.7 cells and acute lung injury in mice by activating the Nrf2/HO-1 pathway and by inhibiting the NF-κB pathway (Wang et al., 2021). Furthermore, some ingredients had been proved to inhibit the inflammatory effectively. For example, evofolin B had been proved to exhibit potent inhibitory activities on human neutrophil O_2_ generation (Chen et al., 2009). Quercetin was reported to protect mice from LPS-induced sepsis by inhibiting TNF-α and IL-1β expressions, NF-κB activation, and apoptosis (Wei et al., 2018) and proposed to prevent myocardial dysfunction through the TLR4/NF-κB signaling pathway during sepsis (He et al., 2020). Paeonol was reported to have protective effects on endotoxin-induced kidney injury by attenuating inflammatory responses and suppressing the TLR4 and NF-κB signaling pathway (Fan et al., 2016), to improve immune response during sepsis by promoting phagocytosis of macrophages through confining HMGB1 to the nucleus (Miao et al., 2020) and protecting the kidneys of sepsis mice through attenuating inflammation by targeting HMGB1 by upregulating miR-339-5p, which was a cancer-related molecule and could participate in multiple cell processes, such as proliferation, migration, and invasion (Jansson et al., 2015; Mei et al., 2019).

### 3.10 KEGG Enrichment Analysis of Critical Functional Ingredients Group Targets

**FIGURE 8:**
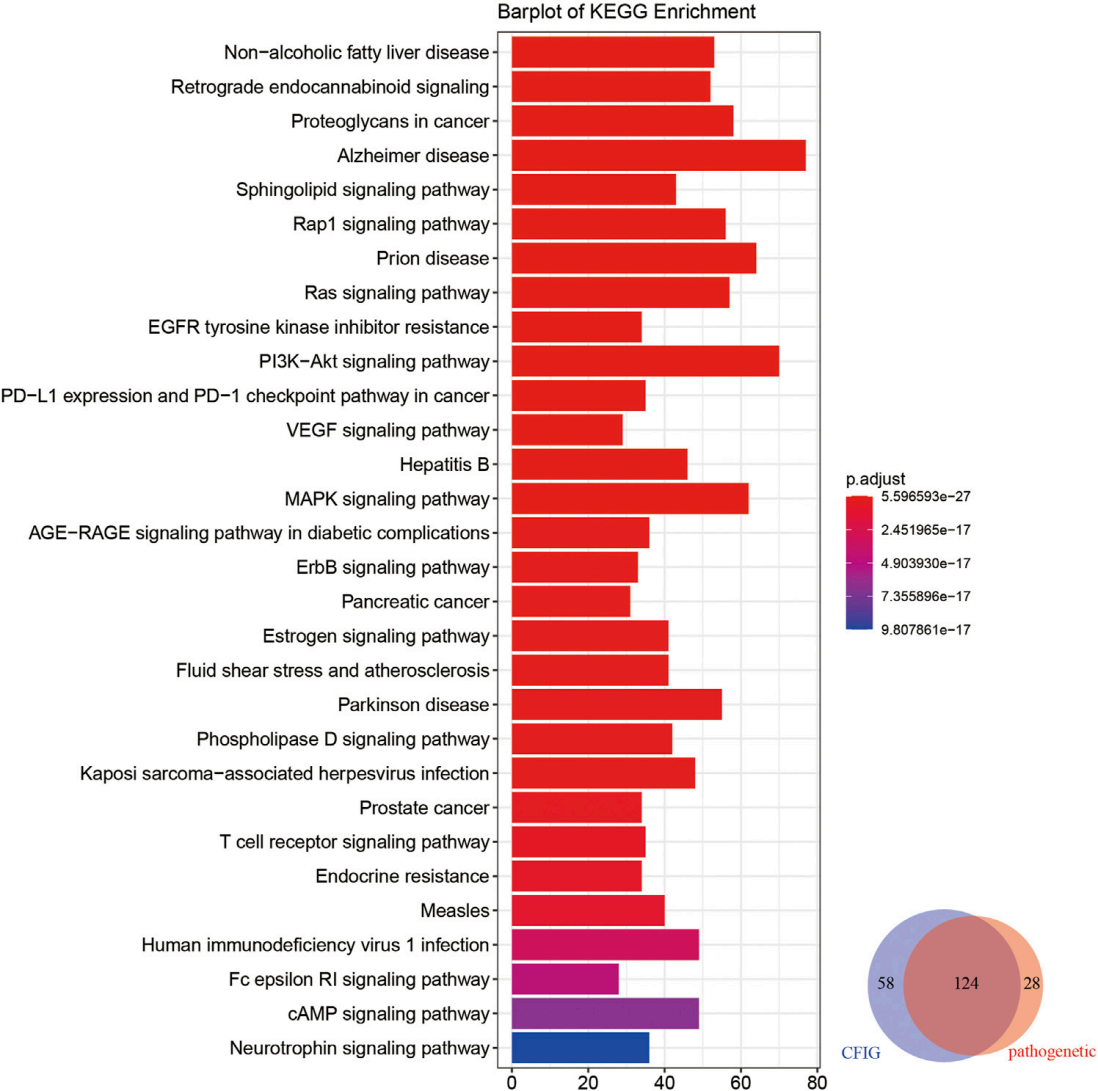
KEGG enrichment analyses of the targets of the CFIG. The color change represents the significance of the enrichment of genes.

For further exploring the potential mechanism of the effect of XBJI in treating sepsis, we conducted a pathway analysis to CFIG targets. The number of CFIG targets enriched pathways was 182 (*p* < 0.05) which could cover 81.58% of the pathogenetic genes enriched pathways (Figure 8).

Based on CFIG targets annotations, some signaling pathways which could not be clearly found out in pathogenetic gene annotations and ingredient target annotations were signed out, which had been reported to be closely related with sepsis according to literatures, such as nonalcoholic fatty liver disease (NAFLD) (hsa04932), retrograde endocannabinoid signaling (hsa04723), proteoglycans in cancer (hsa05205), the sphingolipid signaling pathway (hsa04071), and the Ras signaling pathway (hsa04014).

According to some literatures on NAFLD, some immune-related loci associated with NAFLD could exhibit some level of pleiotropy influencing sepsis with genes of CD14, IL-6, MIF, TLR4, and TNF (Sookoian and Pirola, 2019). NAFLD was reported to upregulate 11β-HSD1, 11β-HSD2, and G6PD, thereby enhancing the production of corticosterone, which could contribute to combat sepsis shock (Huang et al., 2020). Retrograde endocannabinoid signaling could effect the secretion of endocannabinoids, which is a lipid-based retrograde messenger with the effect of regulating the strength of neuron communications and regulating the blood–brain integrity in the restriction of systemic inflammation, such as septic encephalopathy (Kasatkina et al., 2021). A latest study showed that a high abundance of miRNA in community-acquired pneumonia (CAP), a major cause of sepsis, revealed a significant overrepresentation for proteoglycans in cancer, demonstrating that some small non–coding RNA might be related to CAP (Khan et al., 2021). Before this study, Sdc1 had been proved to be a member of the syndecans, which belong to the family of type I transmembrane heparan sulfate proteoglycans in vertebrates and are related to sepsis (Holzmann et al., 2018; Ikeda et al., 2018). The results of CFIG target annotations showed that our model in selecting CFIG was useful in uncovering the potential mechanism of XBJI in treating sepsis.

**FIGURE 9:**
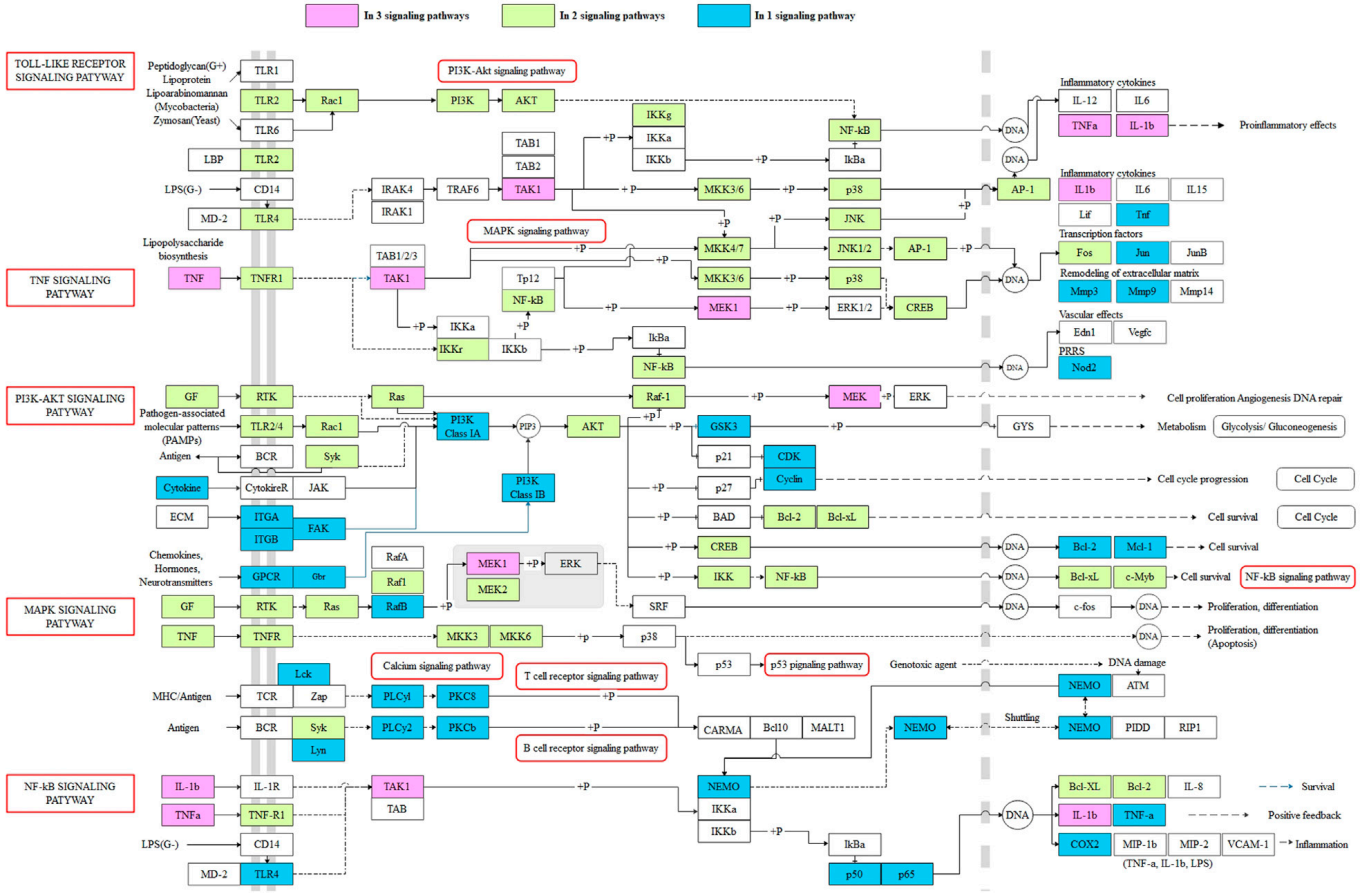
The comprehensive pathway of the targets of the CFIG. Pink represents the CFIG targets or proteins enriched in three pathways, green represents the CFIG targets or proteins enriched in two pathways, blue represents the CFIG targets or proteins enriched in one pathway, and the signaling pathways are marked by red frames.

In order to further explore the potential mechanism of XBJI in treating sepsis, we constructed a comprehensive pathway with five signaling pathways, including MAPK (hsa04010), NF-κB (hsa04064), PI3K-Akt (hsa04151), TNF (hsa04668), and TLR (hsa04620) (Figure 9). These five signaling pathways repeated in the signaling pathway of KEGG enrichments of pathogenetic genes, targets of ingredients, and targets of the CFIG, indicating that these four signaling pathways might be important in XBJI in treating sepsis.

In this comprehensive pathway, some proteins could exist in more than two signaling pathways. For example, TAK1, TNF-α, and IL-1β could exist in three signaling pathways: TLR, TNF, and NF-κB; MEK1 could exist in three signaling pathways: TNF, MAPK, and PI3K-Akt. Most of these proteins had been reported to be closely associated to sepsis and anti-inflammatory responses. For example, 1) TAK1: it was proved to play important roles in the inflammatory response mediated by TLR4, which had been regarded as a method in controlling sepsis (Opal et al., 2013; Chen et al., 2015). It was a signal transducer upstream of p38 MAPK and NF-κB, while the inhibition of the interaction between TAK1 and TAB1 could attenuate TAK1-mediated MAPK activation, thereby alleviating sepsis-induced multiple organ dysfunction in mice (Zhang et al., 2020). Some other factors, such as the IKK complex, p38, JNK, and EKR, could also be functionalized and phosphorylated by activated TAK1, NF-κB, and MAPK signaling pathways, thus being activated afterward (Ajibade et al., 2013). Some studies have proved that the activation of TAK1 could lead to the activation of IKK complex—NF-κB and MAPKs, playing as an essential signaling factor of both NF-κB and MAPK signaling pathways (Kawasaki and Kawai, 2014; Seumen et al., 2021). A latest study showed that TAK1 could be targeted by ubiquitin-specific peptidase 18, negatively regulating and inhibiting LPS-induced sepsis (Hu et al., 2021). 2) TNF-α: This is a typical pro-inflammatory factor, which could promote T cells to further produce a large number of inflammatory mediators, which could reduce the body's immune function and destroy the barrier function of various tissues and organs, leading to the spread of systemic inflammatory response (Kamisoglu et al., 2015). It has been considered to be one of the links in regulating apoptosis (Zhao et al., 2015). Some latest studies showed that the expression level of TNF-α was significantly higher in LPS-induced H9c2 cardiomyocytes, suggesting that the expression of NF-κB/TNF-α might be related to inflammatory response and apoptosis of H9c2 cells during sepsis (Zheng et al., 2020). The level of TNF-α was reported to be increased significantly in LPS-stimulated BMDMs (Liu et al., 2020). Comparatively, TNF-α could be reduced by baicalin (MOL002776) in the blood of sepsis mice (Chen et al., 2016) and was reduced by beta-sitosterol [MOL000358 (CS, DG, and HH)] (Kasirzadeh et al., 2021), caffeic acid [MOL000223 (CX, DS, and HH)] (Chen et al., 2019). A Chinese formula, RDN, had been proved to ameliorate LPS-induced acute lung injury by downregulating TNF-α (Yang et al., 2021). 3) IL-1β: This is also a typical pro-inflammatory factor, which could promote T cells to further produce a large number of inflammatory mediators, which could reduce the body's immune function and destroy the barrier function of various tissues and organs, leading to the spread of systemic inflammatory response (Kamisoglu et al., 2015). Comparatively, it was proved to be downregulated by RDN and to ameliorate LPS-induced acute lung injury (Yang et al., 2021). It was indicated to be decreased by CX (Lv et al., 2012) and reported to be reduced by baicalin (MOL002776) in the sera of bacterial infected mice, significantly, thereby improving mouse survival in bacterial sepsis (Li et al., 2017), and to have been reduced by caffeic acid [MOL000223 (CX, DS, and HH)] in LPS-stimulated macrophages (Chen et al., 2019). IL-1β was also reported to be attenuated by paeoniflorin (MOL001924), a critical ingredient of monoterpene glycoside in CS and XBJI (Wang et al., 2020), thereby improving myocardial function in septic mice (Zhai and Guo, 2016), and reported to be inhibited by quercetin (MOL000098), thereby protecting mice from LPS-induced sepsis apoptosis (Wei et al., 2018); it was also reported to be decreased by ferulic acid (CIS) (MOL000389), an ingredient in DG, thereby positively modulating the inflammatory response to septic liver injury (Cao et al., 2021). 4) MEK: this is a key protein in the MEK signaling pathway, of which it could inhibit apoptosis induced by LPS (Chen et al., 2020). The upregulated phosphorylation of MEK1 was reported to stimulate the syntheses of TLR2 and surfactant protein-A (SP-A) in human alveolar epithelial A549 cells (Wu et al., 2011), where TLR2 existed in human pulmonary epithelial cells and participated in the response to lung injury (Droemann et al., 2003; Slevogt et al., 2008), and SP-A could help the lungs defend bacterial infections (LeVine et al., 2000). Some studies showed that MEK1 could participate in the activation of NADPH oxidase, which was a multi-ingredient enzyme with a function of generating oxygen-dependent antimicrobial arsenals, thereby improving the neutrophil immune function during sepsis (Huber-Lang et al., 2002).

Furthermore, 23 proteins could exist in two pathways in the comprehensive pathway, such as AKT, NF-κB, and p38. Most of these proteins were proved to be related with sepsis. The comprehensive pathway proved that the CFIG of XBJI could affect sepsis by multigenes and multichannel biological processes, suggesting that we need to consider the interactions of targets and ingredients when treating sepsis, and also indicating that our model could be used in selecting the CFIG of XBJI in treating sepsis effectively.

### 3.11 Experimental Validation *In Vitro*

For selecting some potential ingredients which could be effectively used in the *in vitro* experiment, we conducted molecular docking by 59 ingredients with 3D conformer and 160 protein structures responding to 56 genes in comprehensive pathways and obtained 86,475 binding relationships in the docking results (Supplementary Table S8).

**FIGURE 10:**
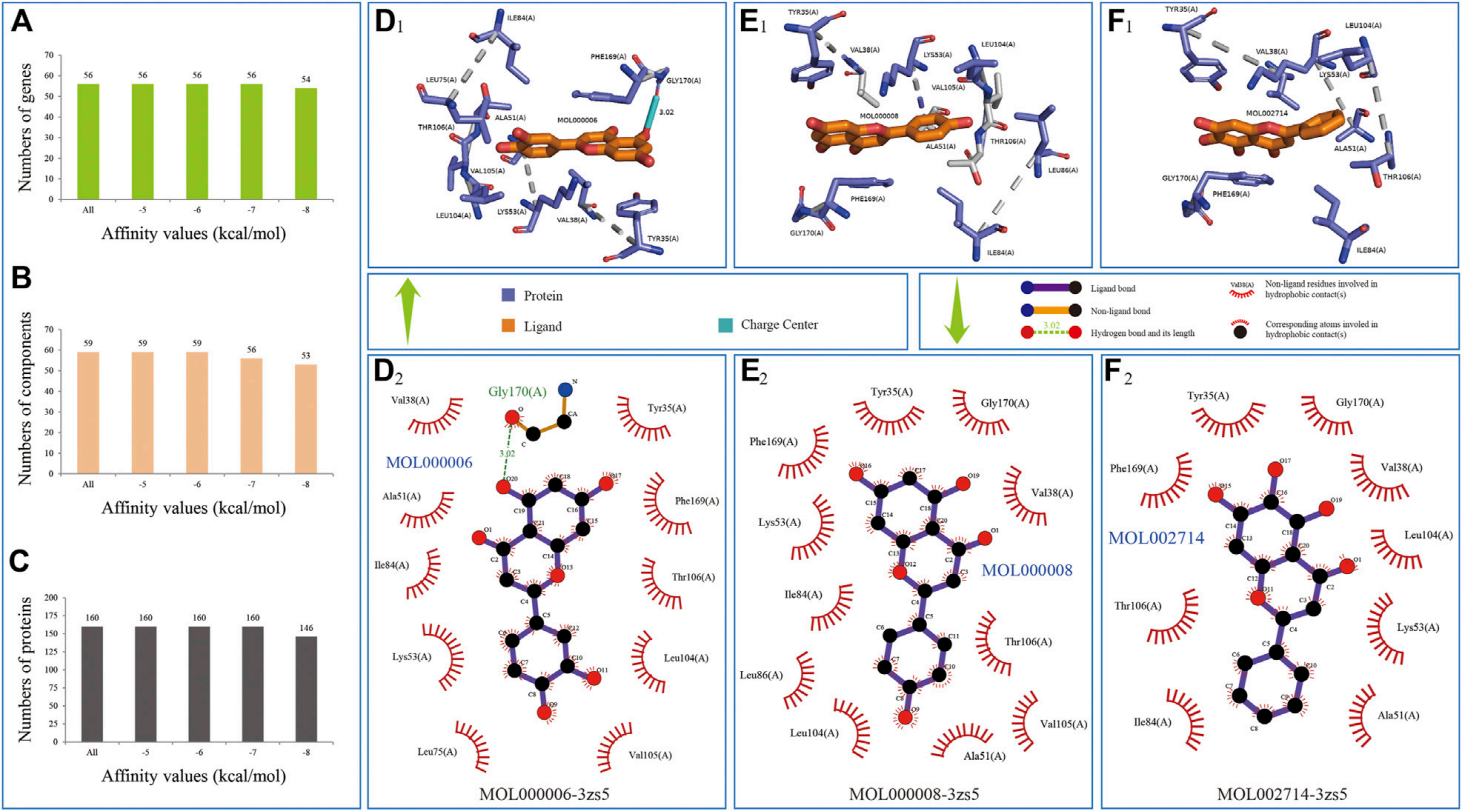
Three binding relationships in docking predictions. Ingredient is colored by elements in yellow; the combined location between ingredient and protein colored by elements in green and blue **(D**_**1**_ **-F**_**2**_ **)** represent the bindings of MOL000006-3zs5, MOL000008-3zs5, and MOL002714-3zs5, respectively.

In the molecule docking, the effectiveness of the interactions between proteins and ligands could be evaluated by the binding energy, such that a lower value of affinity represented better binding energy (Fu et al., 2018; Elhenawy et al., 2019; Cui et al., 2020). The information of binding relationships divided with binding affinity values showed that all the 59 ingredients of the CFIG could bind the 56 genes of the comprehensive pathways, whose affinities were equal or lower than −6 kcal/mol, indicating that the 160 proteins searched from PDB could well represent the effectiveness of the 56 genes in the comprehensive pathways, and confirming that the ingredients of the CFIG could well target the proteins involved in the comprehensive pathways (Figures 10A–C). Three ingredients used in the *in vitro* experiments showed good combinations with proteins. Apigenin (MOL000008) could bind the best with protein structure 3zs5 responding to gene MAPK14 with a binding affinity of −10.70 kcal/mol, while baicalein (MOL002714) and luteolin (MOL000006) could bind the best with protein structure 3zs5 responding to gene MAPK14 (with a binding affinity of −10.40 and −10.40 kcal/mol, respectively) (Figure 10D_1_–F_2_).

Among the 86,475 binding relationships, the ingredient MOL000286 could bind the best with protein structure 3ri1 responding to gene FGFR2 with a binding affinity of −11.50 kcal/mol (Supplementary Figure S2A_1_–A_2_), followed by binding relationships of MOL007111-3zs5 (MAPK14, −11.20 kcal/mol), MOL000286-3iw4 (PRKCA, −11.20 kcal/mol), MOL000357-3iw4 (PRKCA, −11.20 kcal/mol), MOL007154-6q7b (EPHA2, −11.10 kcal/mol), MOL007068-3zs5 (MAPK14, −11.10 kcal/mol), and MOL004495-5ikr (PTGS2, −11.10 kcal/mol) (Supplementary Figure S2B_1_–G_2_). We analyzed in-depth the binding relationships whose binding affinity values were equal to or lower than −6.00 kcal/mol and obtained 52,276 bindings, including 56 genes, 59 ingredients, and 160 proteins, and focused on two questions: 1) which ingredient could bind the most number of genes and 2) which genes could bind the most number of ingredients. The results were as follows: 1) a total of 26 ingredients could bind 56 genes, eight ingredients could bind 55 genes, and 3 ingredients could bind 54 genes, indicating that CFIG could bind the genes in the compressive signaling pathway effectively. Among these 26 ingredients related with 56 genes, and according to the average affinities of each ingredient, three ingredients had the highest value of binding affinities and were COM12, ZINC8234227 MOL004495, and ZINC56874950 MOL002764. The average affinities of these three ingredients were −7.87, −7.85, and −7.64 kcal/mol. The optimal docking bindings of these three ingredients were COM12-5f1a (PTGS2, −10.70 kcal/mol), MOL004495-5ikr (PTGS2, −11.10 kcal/mol), and MOL002764-5f19 (PTGS2, −10.90 kcal/mol) (Supplementary Figure SG_1_–G_2_). 2) Gene MAPK14 could bind the largest number of ingredients with the 59 ingredients, followed by genes EPHA2, FGFR1, and KDR with 58 ingredients, whose average affinities were −7.43, −7.86, −7.28, and −7.32 kcal/mol, respectively. The optimal docking bindings of these four genes were MOL007111-3zs5 (MAPK14, −11.20 kcal/mol) (Supplementary Figure SB_1_–B_2_), MOL007154-6q7b (EPHA2, −11.10 kcal/mol) (Supplementary Figure SE_1_–E_2_), MOL004495-5ew8 (FGFR1, −9.80 kcal/mol) (Supplementary Figure SJ_1_–J_2_), MOL000286-3vhe (KDR, −10.30 kcal/mol) (Supplementary Figure SK_1_–K_2_), and MOL004495-3vhe (KDR, −10.30 kcal/mol) (Supplementary Figure SL_1_–L_2_). These above results indicated that the CFIG could effectively bind with the proteins involved in the comprehensive pathway and is worthy to be studied further for effects in treating sepsis. Here, three ingredients, apigenin, baicalein, and luteolin, were selected into *in vitro* experiments.

**FIGURE 11:**
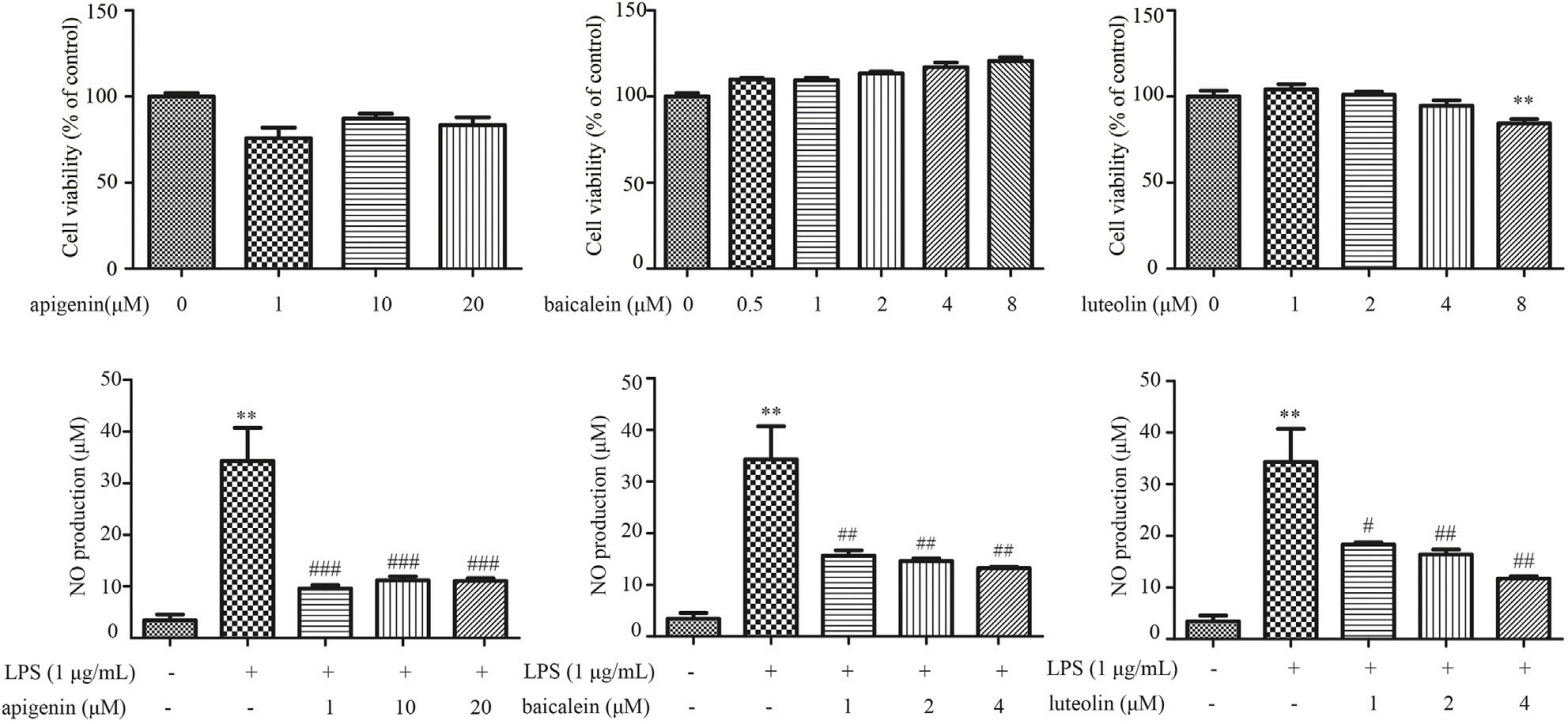
The effects of apigenin **(A,D)**, baicalein **(B,E)**, and luteolin **(C,F)** on the cell viability and suppressing LPS-induced NO in RAW264.7 cells. Data are expressed as mean ± SD (n = 3), ^**^ *p* ＜ 0.01 vs control group alone, ^#^ *p* ＜ 0.05 vs control group alone, ^##^ *p* ＜ 0.01 vs control group alone, ^###^ *p* ＜ 0.001 vs control group alone.

LPS-induced RAW264.7 cells model was utilized to assess the potential anti-inflammatory effect of pivotal ingredients *in vitro*. The results of cell viability showed that apigenin at 1–20 μM, baicalein at 0.5–8 μM, and luteolin at 1–4 μM had no obvious cytotoxicity to RAW264.7 cells (Figures 11A–C). As shown in Figure 11D–F, the level of NO production was markedly increased after LPS treatment, and apigenin (1–20 μM), baicalein (1–4 μM), and luteolin (1–4 μM) significantly decreased the level of NO, which suggests that apigenin, baicalein, and luteolin inhibited NO production in LPS-induced RAW264.7 cells.

## 4 DISCUSSION

TCM plays an important role in treating disease with a systematical theory of that, ingredients of botanical drugs composed to be a formula, and different formula can be used in different disease. In the study of formulas, the main purpose of formula optimization is to reduce the non–pharmacological factors and improve the curative effects (Yang et al., 2021). Nowadays, many formulas are being used in clinical treatments and explored for their mechanisms, such as XBJI. Finding out their critical ingredients and making clear certain effects of the critical ingredients are important in formula optimizations.

In this study, the main purpose was to find out about the CFIG of XBJI in treating sepsis and find a method in selecting the CFIG. Based on the philosophical view that “Chinese medicine is holistic” in Chinese medicine research, we used traditional network pharmacology and proposed an integrative strategy in selecting the CFIG. Our method emphasis the concept of multi-target regulating of signaling pathways, which is useful for exploring the systematical treating effect of TCM, such as the systematical treating effects of XBJI in treating sepsis. Besides the integrative strategy, we further used docking predictions and *in vitro* verifications to predict the effect of the CFIG in treating sepsis with representation of three ingredients, namely, apigenin, baicalein, and luteolin. In molecular docking, we proved that the CFIG could bind proteins involved in the comprehensive pathways effectively, such as β-amyrin acetate (MOL000286), isotanshinone II (MOL007111), sitogluside (MOL000357), tanshinone IIa (MOL007154), przewaquinone B (MOL007068), and tigogenin (MOL004495) (Supplementary Figure S2). These ingredients showed good potential contributions to the treatment of sepsis by the CFIG (Figure 7) and were directly reported to benefit the treatment of sepsis or relate to anti-inflammatory, anticancer, etc. For example, tanshinone IIa was detected in the Qiang-Xin 1 formula, which could be used in treating sepsis-induced cardiac dysfunction and predicted to regulate the productions of intracellular NO, calcium, and mitochondrial ROS (Jin and Li, 2013; He et al., 2020). It was reported to have anticancer effects through the PI3K/Akt/mTOR signaling pathways and present multiple pharmacological activities, such as cardioprotection, neuroprotection, vascular function protection, anticancer, and other such activities (Shang et al., 2012; Su and Chiu, 2016; Fang et al., 2020). β-amyrin acetate was reported to have anti-allergic and anti-inflammatory effects (Akihisa et al., 2010; Thabet et al., 2018) and to be antiproliferative against cancer cells (Zehra et al., 2019). Tigogenin and its conjugates, neoglycosides and analogues, exhibited synthesis, antitumor, and immunomodulating activities (Li et al., 2018; Michalak et al., 2020). As we know, the dose–effect relationship of the CFIGs and their functions is important. It had been pointed out that the doses of XBJI used in clinics might be too low to reach the effective concentrations of the key ingredients, thereby failing to exert an antiseptic effect (Li et al., 2016). The experiments in the septic shock model also supported this theory in that there were no insignificant impacts of 2 ml/kg XBJI on the differentiation of CD4 T cells, pro-inflammatory cytokine production, neutrophil infiltration in organs, and survival, while the treatment of 18 ml/kg for 5 days could significantly improve the survival, and the treatment of a 6-ml/kg dose had a trend of improving survival without statistical significance (Chen et al., 2018). In our study, results in the *in vitro* verifications also showed that there was a dose–effect relationship of the critical functional ingredients in treating LPS-induced RAW264.7 cells. Baicalein (2 and 4 μM) could significantly decrease the level of NO which was lower than the level of NO in the treatment with a concentration of 1 μM, and these results were similar to luteolin. At the same time, it has been found that high concentrations of some ingredients might have an adverse effect on cell survival because 8 μM of luteolin was found to induce cell viability of RAW264.7 cells significantly in our study. For these results or phenomenon, we speculate that the composition of the CFIG in XBJI and the dose of each ingredient play the same important role in the treatment of sepsis. In our study, we proposed a new model to select the CFIG of XBJI. The results of both docking predictions and *in vitro* verifications proved that the CFIG of XBJI played an important role in treating sepsis and further confirmed that our strategy in selecting the CFIG was accurate and effective. Due to hundreds of ingredients in the TCM formula, we hoped to use the CFIG selection model to figure out the critical function ingredients, which is within the tolerable range of verification and can be used for dose selection in *in vitro* verification.

Based on the basics of some studies, which had provided a powerful tool in exploring the compatibility and mechanism of the TCM formula with network pharmacology, we further proposed an integrative strategy and got the CFIG of XBJI in treating sepsis under the combinations with pathogenetic genes and ingredients of XBJI closely. Among the CFIG, targets of the top nine ingredients in the CFIG (ferulic acid, ethyl ferulate, evofolin B, quercetin, guanosine, methyl tricosanoate, paeonol, luteolin, and apigenin) of XBJI could cover 50.63% of the key response proteins (Figure 7), providing a strong reference for other formulae optimization. At the same time, many cytokines or proteins were found to be the targets of the ingredients in the CFIG (Supplementary Table S9). For example, proteins in the TLR family and TLR signaling pathway have been proved to play important roles in the cytokine storm of sepsis (Kumar, 2020b). TLR2 and TLR4 were found to have more dynamic expression on neutrophils than on monocytes in sepsis (Salomão et al., 2008). The expressions of these two proteins were found to increase in the kidneys and intestine of sepsis mice, indicating that these four cytokines could influence the effect of pro-inflammatory response during infection (Krivan et al., 2019). TLR4 could control bacterial clearance and induce pro-inflammatory immune response during bacterial sepsis (Deng et al., 2013). Among the ingredients in the CFIG, TLR2 was the target of MOL005368, MOL000012, MOL001494, MOL002883, MOL001838, MOL001399, MOL002759, and MOL002677; TLR4 was the target of MOL002677. TLR9 was the target of MOL000389 and COM10. IL-1β was a typical pro-inflammatory factor, which could promote T cells to further produce a large number of inflammatory mediators (Kamisoglu et al., 2015), and the target of MOL000286, one of the ingredients in the CFIG. IL-2 was the target of MOL000098, MOL000874, MOL000006, MOL000008, MOL002714, MOL005043, COM14, COM12, MOL000357, MOL002141, MOL000286, MOL007006, and MOL004495. NF-κB could promote the transcription of some pro-inflammatory cytokines, including IL-1β, IL-12, and TNF-α (Hotchkiss et al., 2016). Some pro-inflammatory cytokines, such as IL-6 and TNF-α, could be activated through the NF-κB signaling pathway and could be regulated by XBJI (Chen et al., 2018). Furthermore, the expression of some cytokines in NF-κB signaling, such as CD14, CXCL2, and Ptgs2, were also reported to be inhibited by XBJI (Wang et al., 2020). In our study, NFKB1 was the target of many ingredients in the CFIG, including MOL000389, COM10, MOL007022, MOL000874, MOL000223, COM17, MOL007116, COM16, MOL007050, and MOL001942. TNF-α, a pro-inflammatory cytokine, could be promoted to transcript through the NF-κB and MAPK signaling pathway, thereby activating the cytokine storm (Hotchkiss et al., 2016; Yang et al., 2021). It was the target of MOL001494, MOL002883, and COM12. Some reports had showed that some signaling pathways could mediate the development of sepsis. For example, the JAK/STAT signaling pathway could mediate myocardial injury of septic rats (Jin et al., 2020); the JAK2/STAT3 signaling pathway could promote BBB impairment in septic rats (Chen SL. et al., 2020); PI3K-Akt signaling pathway could attenuate apoptosis and improve the survival in animal models with sepsis (Liu et al., 2019; Mizuta et al., 2020). Among the targets of the ingredients in the CFIG, STAT1 was the target of MOL000874 and MOL000357; STAT2, STAT3, and STAT4 were the targets of MOL000357; AKT1 was the target of MOL000098 and MOL002771. These five proteins, STAT1, STAT2, STAT3, STAT4 and AKT1 exit in JAK/STAT, JAK2/STAT3 or PI3K-Akt signaling pathway. These results indicate that the CFIG selected from our proposed model was the potential effective ingredients in XBJ during the cytokine storm of sepsis. Furthermore, we found some KEGG signaling pathways which were just discovered and studied in sepsis in recent years, such as nonalcoholic fatty liver disease (hsa04932) (Sookoian and Pirola, 2019; Huang et al., 2020), retrograde endocannabinoid signaling (hsa04723) (Kasatkina et al., 2021), and proteoglycans in cancer (hsa05205) (Holzmann et al., 2018; Ikeda et al., 2018; Khan et al., 2021) (Figure 10). These signaling pathways may have the potential and be important in treating sepsis with XBJI and are worthy to be studied further.

In our study, *in vitro* experiments were further used to value whether the ingredients selected were useful in treating sepsis at the experimental level. The three ingredients, apigenin, baicalein, and luteolin, proved to be effective in LPS-induced RAW264.7 cells with the evaluation indicators of the NO level. According to some reports, these three ingredients have also been proved to reduce the NO level at other concentrations in LPS-stimulated RAW264.7 cells. For example, apigenin could reduce the NO level significantly at the concentrations 60, 80, and 100 μM (Park et al., 2020); baicalein could reduce the NO level significantly at the concentration of 100 μM (Fan et al., 2013); luteolin could reduce the NO level significantly at the concentrations 25, 100, and 200 μM (Zhou et al., 2020). Furthermore, these three ingredients were also proved to be effective in LPS-induced RAW264.7 cells with the evaluation indicators of IL-1β and TNF-α. Some results in these reports are as follows: 1) the effect of apigenin on RAW264.7 cells showed that 100 μM apigenin could significantly inhibit the IL-1β expression and could ineffectively increase TNF-α (Park et al., 2020). 2) Some experiments with RAW264.7 cells showed that baicalein could significantly inhibit the levels of IL-1β and TNF-α at the concentrations 0.1, 1, and 10 μM (Fan et al., 2013) and significantly inhibit the level of TNF-α at the concentrations 5, 20, and 50 μM (Zhang et al., 2021). 3) Based on the experiments with RAW264.7 cells, luteolin had been proved to significantly inhibit the level of TNF-α at the concentrations 1, 2, 4, and 8 μM and at 5, 20, and 80 μM (Guo et al., 2021). Furthermore, it had been proved to significantly inhibit the level of IL-1β and TNF-α at the concentrations 2, 25, 100, and 200 μM (Zhang et al., 2018; Zhou et al., 2020). These reports and the results of our study further prove that our model was effective and accurate in selecting the CFIG of XBJI in treating sepsis.

Besides the *in vitro* experiments in our study, some ingredients in the CFIG had also been found to benefit the treatment of sepsis. For example, caffeic acid (MOL000223) could inhibit the production and expression of pro-inflammatory cytokines in LPS-induced RAW 264.7 cells, such as TNF-α, IL-1β, and IL-6, at the concentrations 25, 50, and 100 μM (Chen et al., 2019). Crysophanol (MOL001729) could inhibit the expressions of IL-1β, NF-κB, NF-kB P65, and PPAR-γ in LPS-induced RAW 264.7 cells at the concentration of 15 μM, while PPAR-γ had been reported to capably inhibit LPS-induced inflammatory responses in macrophages by suppressing NF-κB activation to inhibit the release of pro-inflammatory cytokines (Wen et al., 2018). Based on the mouse model of acute liver injury (ALI) induced by cecal ligation and perforation (CLP) and a cell model of ALI stimulated by LPS, pretreatment with ferulic acid (MOL000389) had proved to significantly reduce liver/body weight ratio, decrease the activities of MPO, AST, and ALT, and alleviate the inflammatory responses and improve CLP-induced histopathological changes in the liver. It was also proved to dose-dependently increase the viability of RAW264.7 cells and decrease the levels of IL-10 and IL-1β (Cao et al., 2021). Furthermore, some ingredients in the CFIG were also proved to be anti-inflammatory even though they had not been studied in sepsis. For example, galuteolin (COM12) had been proved to have anti-inflammatory activities with significant inhibition of the expressions of CRP, JNK2, and NO to the LPS-induced RAW264.7 cells (Mao et al., 2017). β-amyrin acetate could significantly inhibit the expression of TNF-a and IL-6 in LPS-induced RAW264.7 cells (Ding et al., 2009). Gamma-tocotrienol (MOL002684) could significantly inhibit IL-6 or NO expression in LPS-induced RAW264.7 cells and had anti-inflammatory activities by inhibiting NF-κB and C/EBPs activation in macrophages (Yam et al., 2009; Wang and Jiang, 2013). These ingredients might have the potential effect on the treatment of sepsis and be worth studying further.

However, there were some limitations in this study. Firstly, more ingredients from the CFIG should have been selected for validating the reliability of our method and model. Secondly, the precise mechanisms which were uncovered by the maximum targeting weight model need further validation. Finally, our algorithm which was used in the undirected network ignored the activation or inhibition effects of the genes.

On the whole, we proposed a reverse optimization model based on the association of pathogenetic genes and ingredient targets to improve the accuracy on decoding the CFIG of XBJI, providing reference for the optimization and mechanism analysis of the formula in TCM.

## Data Availability

The original contributions presented in the study are included in the article/Supplementary Material; further inquiries can be directed to the corresponding authors.

## Glossary

AST: aspartate aminotransferase
BMDMs: bone marrow–derived macrophages
CAP: community-acquired pneumonia
CCK-8: Cell Counting Kit-8
CFIG: critical functional ingredients group
CKMB: creatine kinase-MB
CS: *Paeonia lactiflora* Pall. (Chishao)
CX: *ligusticum striatum* DC. (Chuanxiong)
DG: *Angelica sinensis* (Oliv.) Diels (Danggui)
DMEM: modified Eagle's Medium
DS: *Salvia miltiorrhiza* Bunge (Danshen)
FBS: fetal bovine serum
FRS: functional response space
HH: *Carthamus tinctorius* L. (Honghua)
HMGB1: high mobility group box protein B1
ICP: Ingredients–Contribution–Proportion
I-T: ingredients–targets
JNK: C-Jun N-terminal kinase
LPS: lipopolysaccharide
MIF: migration inhibitory factor
MODS: multiple organ dysfunction syndrome
MP: membrane potential
NAFLD: nonalcoholic fatty liver disease
NFAT: nuclear factor of activated T cells
NFATC2: nuclear factor of activated T cells 2
PAF: platelet-activating factor
RDN: Re-Du-Ning injection
RIP-1: receptor interacting protein-1
ROS: reactive oxygen species
RQKT: Qingkailing Injection
SEA: Similarity Ensemble Approach
SFI: Shenfu injection
SIRS: systemic inflammatory response syndrome
SP-A: surfactant protein-A
sTREM-1: soluble triggering receptor expressed on myeloid cells-1
TCM: traditional Chinese medicine
TLR: Toll-like receptor
TLRs: Toll-like receptors
TNF: tumor necrosis factor
TNFR1: TNF receptor 1
TNFR2: TNF receptor 2
TRAF-2: TNF receptor–associated factor-2
UETs: un-optimized effective targets
XBJI: Xuebijing injection
