# Supplementary figures and images for: Detecting Critical Functional Ingredients Group and Mechanism of Xuebijing Injection in Treating Sepsis
Source: Front Pharmacol. 2021 Dec 6;12:769190. doi: 10.3389/fphar.2021.769190 (PMC8687625; doi:10.3389/fphar.2021.769190)

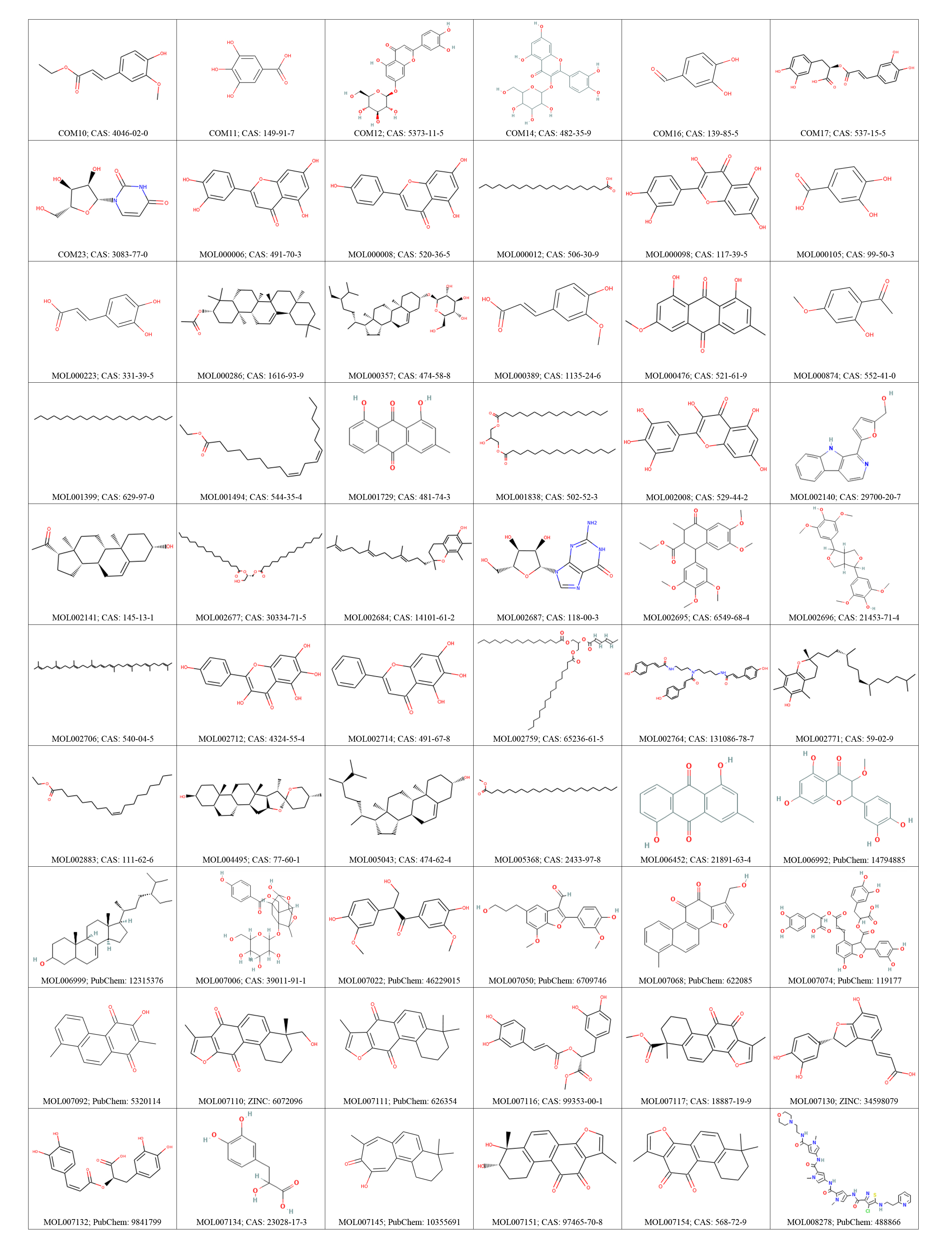

Supplement: Supplementary file 4 [file Image1.JPEG]

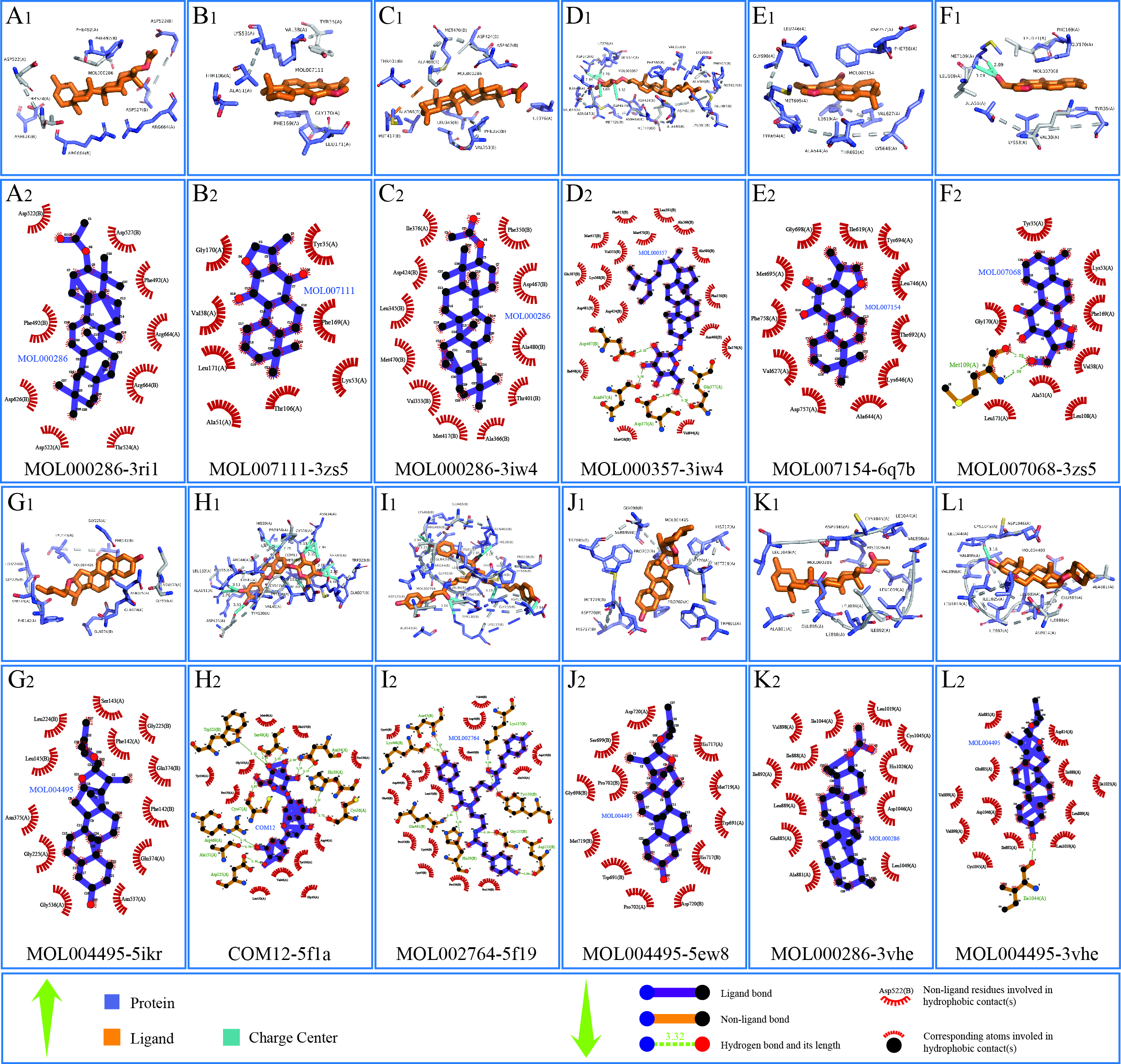

Supplement: Supplementary file 5 [file Image2.JPEG]
